# On quantification and maximization of information transfer in network dynamical systems

**DOI:** 10.1038/s41598-023-32762-7

**Published:** 2023-04-05

**Authors:** Moirangthem Sailash Singh, Ramkrishna Pasumarthy, Umesh Vaidya, Steffen Leonhardt

**Affiliations:** 1 Electrical Department, IIT Madras, Chennai, India; 2 Mechanical Department, Clemson University, Clemson, USA; 3 Chair for Medical Information Technology, RWTH Aachen University, Aachen, Germany

**Keywords:** Information technology, Computational science, Applied mathematics, Neural circuits

## Abstract

Information flow among nodes in a complex network describes the overall cause-effect relationships among the nodes and provides a better understanding of the contributions of these nodes individually or collectively towards the underlying network dynamics. Variations in network topologies result in varying information flows among nodes. We integrate theories from information science with control network theory into a framework that enables us to quantify and control the information flows among the nodes in a complex network. The framework explicates the relationships between the network topology and the functional patterns, such as the information transfers in biological networks, information rerouting in sensor nodes, and influence patterns in social networks. We show that by designing or re-configuring the network topology, we can optimize the information transfer function between two chosen nodes. As a proof of concept, we apply our proposed methods in the context of brain networks, where we reconfigure neural circuits to optimize excitation levels among the excitatory neurons.

## Introduction

The cause-effect relationships between various events or processes, in which an event contributes to the evolution of another event or a state occurs in different physical^1^, biological^2^’^3^, financial^4^, or technological systems or networks^5^’^6^. Apart from science, causality has been an important topic in contemporary philosophy and its branches, including metaphysics, ontology, and epistemology. In physical systems, Maxwell illustrated with an experiment (Maxwell’s demon)^7^ that reveals the relationship between information and entropy. The experiment showed that the restrictions imposed by the second law of thermodynamics can be relaxed by using information (velocity and positions of the particles) contained in Maxwell’s demon. These notions of information and entropy provide a thermodynamical description of information flows in dynamical systems^8^. In a social network^9^’^10^, information is encoded in the network topology and essential for building reputation, trust, better collaboration, or finding short chains in an extensive social network. In cell biology^2^’^3^, the receptor function relies on precise dynamical communication and coordinated information transfer between the cell surface receptors and the outside world and within the gene networks. In neurological networks^11^, information transfers happen across the synapse by the activity of several neural populations; the dendrites transmit information to the cell body, and the axon transmits information away from the cell body.

The pattern of connections between the proteins or neurons determines how information flows through the gene regulatory networks or neural circuits. During evolution, the gene essentiality changes, and the number of connections between the essential and non-essential genes depends on the ancestral species. Increased interactions among the genes result in transforming non-essential genes into essential genes^12^. Thus knowledge of the ‘wiring’ of these networks helps understand how the collective behaviour contributes to the information flows among the cells. The connectome describes the complete structural wiring diagram of the neurons in the nervous system. Studies show that the changes in the ability to learn and form memories in the nervous system depend on the modification in the synaptic strength through potentiation or depression,^13^’^14^’^15^. An approach to modify synaptic strengths is to reconfigure the wiring by changing the physical connections between the neurons^16^’^17^. Recent evidence has shown that network rewiring is an essential mechanism in learning and neuroplasticity, defined as the ability of the brain to modify the information flows among the neurons in response to intrinsic and extrinsic stimuli^18^’^19^.

Most of the literature around the study of complex dynamical networks focuses mainly on the study of controllability and reachability of nodes and their roles in controlling the network dynamics^20^’^21^. And a few other works focus on identifying the effective connectivity from time series data, such as Fourier-based or polynomial-based interpolation^22^’^23^. These methods are based on interpolation techniques, and the estimation accuracy depends hugely on the chosen basis functions. Various studies on complex networks are specifically focused on the analysis of complex brain networks^24^’^25^’^26^’^27^. The methods to investigate the functional connectivity between brain regions in these works include connectivity models such as structural equation modelling^24^’^25^, dynamic causal modelling^26^, or Granger causality^27^. The structural equation modelling method^24^’^25^ is based on estimating the correlation matrix between the brain regions and is intractable for large networks. Dynamic causal modelling^26^ estimates the connectivity by perturbing the brain’s dynamic system and measuring the response and does not incorporate an information-theoretic measure. Granger causality characterizes the direction of information flow, but it does not quantify the amount of causal inferences. Therefore, in the event of bidirectional causal inferences, Granger causality is difficult to differentiate the relative strengths. The works in all these studies of brain networks focus only on finding the effective connectivity in the brain network. Recently, network scientists have integrated information theory with network theory to study the flow of information in complex networks^28^’^29^. These studies focus mainly on estimating information transfers in stationary random processes. However, in this work, we consider complex dynamical networks with intrinsic stochastic nodal dynamics that can provide accurate estimates of the evolution of information transfers. We model the neurological network based on a dynamic model of the brain (Wilson–Cowan model) and infer the coupling strengths by perturbing the system and finding the phase responses (Phase Response Curve). In this regard, our methods for estimating coupling strengths from neurophysiological time series differ from the methods in^22,23^ where there is no designed perturbation, and the inputs are treated as unknown. We attempt to answer two crucial questions: (i) Is there a way to quantify the information flows among nodes in complex dynamical networks? (ii) What are the effects of changing the network topology on the information transfers among the nodes? Moreover, assuming we have the authority to configure the network topology, can we maximize the information transfer between two predefined nodes? A major distinctive feature of our work, therefore, lies in integrating theories from information theory, graph theory and optimization algorithms to quantify the flow of information between various nodes in complex dynamical networks and finding the optimal topology for maximized information flows.

There are various information-theoretic measures to quantify information flow, such as the time-delayed mutual information^30^, causation entropy^31^, Granger causality^32^ etc. One limitation of these measures is the lack of determining the cause-effect relation or the direction of information flow. Schreiber’s transfer entropy^33^ describes the flow of information between two random processes and provides a directional sense to the information transfer. However, evidence^34^ has shown that transfer entropy may give qualitatively incorrect results, such as imperfect observations of the states, and, as a result, may not always successfully quantify the true information transfers in dynamical systems^35^. Recently, Liang and Kleeman^36^’^37^ formulate the evolution of information transfers in dynamical systems. In our work, we adopt Liang-Kleeman’s formalism of information transfer to measure the flow of information in a network. This formalism has been used to understand causal inference using time series data in large-scale networks^38^ and for identifying sources of instability in network power systems^39^.

To understand the effects of topological changes on the information transfer, we analyze the structural set properties of the information transfer function. Our information transfer function is also closely related to the mutual information^40^, defined as the amount of information obtained about one random variable by observing the second random variable. Solving the maximization of mutual information under a constraint on the marginal distribution has been proven to be NP-Hard^41^’^42^. Maximizing information transfer under edge constraints is a variant of such problems, and we propose algorithms with provable suboptimality bounds for solving such problems. We split the objective function in our maximization problem into two parts: a first term capturing the network topology and a second term capturing the edge weights. Finding the optimal topology problem can be divided into three subproblems : (a) Design Problem: To design a near-optimal topology given the number of nodes and edges (b) Update Problem: To add a fixed number of edges in a given network and (c) Rewiring Problem: Reconfigure a fixed number of edges that maximizes information transfer. The weights of each edge are upper bounded by a positive weight, and a positive real number bounds the total edge weights. A few questions arise naturally, which we will answer in this report. What is the approximation guarantee when the Greedy Algorithm is used in solving the problems? Are there any algorithms that perform close to the Greedy Algorithm while reducing the computational cost? As a computationally cheaper alternative to the Greedy Algorithm, we propose a new algorithm, the ‘Sub-Graph Completion Algorithm’, that performs closely to the Greedy Algorithm while reducing the computational cost by three folds. We also propose a new centrality measure named ‘Information Transfer Edge Centrality’ that quantifies the contributions of edges towards information transfers among nodes in the network. Finally, we apply our proposed algorithms and validate the approximation guarantees in various random networks. We also apply our algorithms to maximize information transfer between two excitatory neurons in a neurological network.

**Figure 1:**
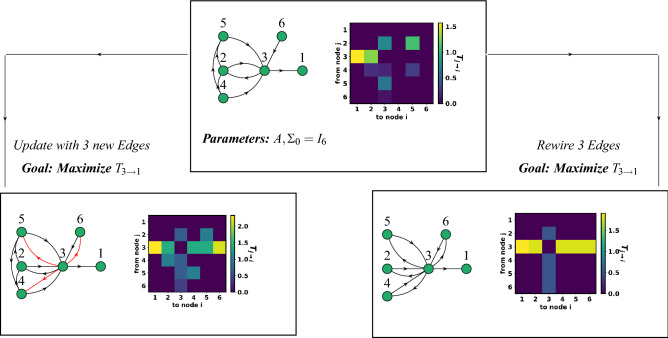
Rewiring network topologies to maximize information transfer from node 3 to node 1. The top figure shows a network of 6 nodes and 9 edges, with zero initial mean, initial covariance, $\Sigma _0 = I_6$, $I_n$ is an identity matrix of order *n*, and $B_1 = 0.1I_6$. The matrix heat map corresponds to the various information transfers among the nodes at $t=10$. The bottom figures show the network topologies which maximize the information transfer using the update and the rewiring techniques.

## Results

### Quantifying the information transfer

To compute the information transfer, we consider a directed network with linear time-invariant stochastic dynamics given by:

1 $$\begin{aligned} dx(t) = Ax(t)dt + B_1dw(t), \end{aligned}$$

where $x(t) \in {\mathbb {R}}^n$ are the nodal states of the network, $w(t)\in {\mathbb {R}}^m$ is a white noise with mean zero and unit covariance and $B_1$ denotes the input noise matrix. The choice for the model is motivated by the fact that we can reduce most real-world oscillatory dynamical networks into phase description models that can be approximated by linear stochastic systems^29^. The model does not incorporate control nodes in the network system and the problem formulation does not require the controllability constraint to maximize the objective function. We assume that the initial states *x*(0) denoted as $x_0$ are drawn from a normal Gaussian distribution $\rho$, with initial mean $\mu _0$ and covariance $\Sigma _0$. Additionally, we assume that there are no self-loops in the considered networks. The transpose of the state matrix, $A^T \in {\mathbb {R}}^{n\times n}$ describes the weighted adjacency matrix. The directed graph is denoted by $\mathcal {G(V,E_A}, w_{{\mathcal {A}}})$, with vertices ${\mathcal {V}} = \{1,2,\ldots n\}$, given by the *n* states, $\mathcal {E_A} = \{(i,j)| i,j \in {\mathcal {V}}\}$ is the edge set, and $w_{{\mathcal {A}}}: \mathcal {E_A} \rightarrow {\mathbb {R}}_+$ is the weight function. The non-zero entries of $B_1$ define how each of the nodes is affected by the white noise. For the linear time-invariant stochastic network model in (1) with *n* random variables and edges ${\mathcal {E}}_{{\mathcal {A}}}$, information transfer from node *j* to *i* at time *t* for $i,j \in \{1,2,3\cdots n\}$, denoted as $T^t_{j\rightarrow i}$ is

2 $$\begin{aligned} T^t_{j\rightarrow i}(\mathcal {E_A}) = -E\bigg [\frac{1}{\rho _i} \int _{{\mathbb {R}}^{n-2}} \frac{\partial f_i~\rho _{\not j}}{{\partial x_i} }\bigg ] = a_{ij}\frac{\sigma ^t_{ij}(\mathcal {E_A})}{\sigma ^t_{ii}(\mathcal {E_A})}, \end{aligned}$$

where $\rho _{\not j}$ denotes the joint distribution of $(x_1,\cdots x_{j-1},x_{j+1},\cdots x_n)$ at time *t*, $\rho _i$ denotes the marginal distribution of state $x_i$,$\rho _{j|i}$ is the conditional probability distribution of $x_j$ given $x_i$ at time *t* and $\sigma ^t_{ij}$ denotes the (*i*, *j*) element of $\Sigma ^t$. The derivations are given in Supplementary Notes 1 and 3. In this work, we consider the case where the network ${\mathcal {G}}$ admits cooperative (i.e., $A(i,j) \ge 0$) interactions among the nodes as negative interactions are not physically meaningful in biological networks and other real-world networks. We shall drop the explicit dependence of $T_{j\rightarrow i}$ on *t*, as maximizing $T_{j\rightarrow i}$ for one time instant maximizes it for all other time instances (Corollary 1.1, Supplementary Note 5). Figure 1 shows our framework for maximizing information transfer from node 3 to node 1 in a given network. In Supplementary note 2, we show the theoretical relationships between Liang-Kleeman’s information transfer, Horowitz’s information, and Schreiber’s transfer entropy.

### Structural analysis of information transfer function

For the directed network, $\mathcal {G(V,E_A}, w_{{\mathcal {A}}})$ associated with the system in (1), we study the structural properties of $T_{j\rightarrow i}$. The domain of $T_{j\rightarrow i}(\mathcal {E_A})$ is the subset of edges, $\mathcal {E_A} \in {\mathcal {E}}$, where ${\mathcal {E}}$ is the set of all possible edges of $|{\mathcal {V}}|$ nodes and the range is a positive real number. It is easy to see from (2) that $T_{j\rightarrow i}(\mathcal {E_A})$ is a function of two set functions $\sigma _{ij}(\mathcal {E_A})$ and $\sigma _{ii}(\mathcal {E_A})$. To maximize $T_{j\rightarrow {i}}$, we need to maximize $\sigma _{ij}$ and minimize $\sigma _{ii}$ concurrently. However, this approach is not feasible as both $\sigma _{ij}$ and $\sigma _{ii}$ are monotone non-decreasing functions of edges (Lemma 1, Supplementary Note 5). Alternatively, we find the set of edges, ${\mathcal {E}}_g \subseteq {\mathcal {E}}$, such that if any edge from ${\mathcal {E}}_g$ is added to $\mathcal {E_A}$, the marginal increase in $\sigma _{ij}$ is greater than the marginal increase in $\sigma _{ii}$. We can formally define the set ${\mathcal {E}}_g$ as

3 $$\begin{aligned} {\mathcal {E}}_g = \{x|\sigma _{ij}(\mathcal {E_A}\cup {x})-\sigma _{ij}(\mathcal {E_A}) > \sigma _{ii}(\mathcal {E_A}\cup {x})-\sigma _{ii}(\mathcal {E_A}); x\in {\mathcal {E}} \} \end{aligned}$$

Thus, it is easy to see from (3) that $T_{j\rightarrow i}$ is a monotone increasing function of the edges in the set ${\mathcal {E}}_g$. To find the elements of ${\mathcal {E}}_g$, we recall the definition of “communicability”^43^ from graph theory. Communicability from node *i* to node *j* in ${\mathcal {G}}(\mathcal {V, {\mathcal {E}}})$, $i,j \in {\mathcal {V}}$, denoted as $c_{ij}$ is defined as the total number of walks of all lengths from node *i* to *j*, weighting walks of length *k* by a factor $\frac{1}{k!}$. It quantifies the ability to exchange messages between two nodes and is given by

4 $$\begin{aligned} \begin{aligned} c_{ij}&= [e^{( {\mathcal {A}}_{0,1})}]_{ij} = {\mathcal {A}}_{0,1} (i,j) + \frac{( {\mathcal {A}}_{0,1})^2}{2!}(i,j) + \cdots \end{aligned} \end{aligned}$$

where ${\mathcal {A}}_{0,1}$ is the structural interconnection matrix of ${\mathcal {G}}$. A walk of length *k* is a sequence of nodes $n_1, n_2, \cdots n_k, n_{k+1}$ such that for all $1 \le l \le k$, $(i_l, i_l+1) \in {\mathcal {E}}$. The relationship between $\sigma _{ij}$ and $c_{ij}$ is given by (Theorem 1, Supplementary Note 5) $\sigma _{ij} \approx \sum ^n_{k=1}c_{ki}c_{kj}, ~~ \sigma _{ii} \approx \sum ^n_{k=1}c^2_{ki}$. Thus, a comparison between (4) and $\sigma _{ij}$ reveals that $\sigma _{ij}$ increases for every incoming path of any length to node *j*, with higher contributions from shorter paths to node *j*. Similarly, $\sigma _{ii}$ increases quadratically with incoming paths to node *i*, with the highest contributions from shortest (direct) paths to node *i*. Therefore, if we fix the in-degree of node *i* to 1 with the only edge to node *i* from node *j*, then any directed paths to node *i* formed by the remaining edges pass through node *j*. As a result, node *j* has shorter directed paths as compared to node *i*, and by definition of communicability, $\sigma _{ij}$ and $\sigma _{ii}$ satisfies the inequality condition in (3). Consequently, if we assume there are no incoming edges to node *i* except from node *j*, then $T_{j\rightarrow i}$ is a monotone non-decreasing function of edges (Theorem 1, Supplementary Note 5). Now, we consider the case when a given network has direct edges to node *i* from nodes other than node *j*. In this case, we avoid adding edges that form directed paths to node *i* but not passing through node *j* for reasons explained earlier. These edges significantly increase $\sigma _{ii}$ while their contribution towards $\sigma _{ij}$ is minimal. Supplementary Fig. 3 shows the structure of the set ${\mathcal {E}}_g$ in the adjacency matrix. The results in this section reveal the relationship between the network structure and the functional pattern, defined by the information transfer function. In the next section, we formally define our problem definitions and propose algorithms to solve the maximization problem. We define the set of possible edges that can be added as the “Ground Set” and is given by ${\mathcal {E}}_g$.

### Finding the optimal topology

We now propose algorithms for solving our optimization problems, namely the design, update, and rewiring problems. The update problem can be considered as a sub-class of design problem since we are adding k edges to existing network topology.

#### Problem 1: design problem

The design problem is to construct a connected network topology with *n* nodes and *k* edges that maximize the information transfer from a predefined node *j* to another predefined node *i*, where $j, i \in \{1,2,\ldots n\}.$ The total edge weight is bounded by $w_{max} \in {\mathbb {R}}^+$. Additionally, the weights of each link, $w_i$ are upper bounded by $w_{ub}$. Our first objective is to find the topology that maximizes $T_{j\rightarrow i}$ by adding minimum edges that ensure the network is at least weakly connected. This topology is a tree network with $n-2$ edges into *j* from the remaining nodes and an edge from node *j* to *i*. We call this the base topology and denote the set of edges by ${\mathcal {E}}_b$. The design problem now is to add $k-n+1$ edges from the ground set ${\mathcal {E}}_g$ to the base topology, that maximizes $T_{j\rightarrow i}$. We then find the optimal edge weights for every new edge. The problem can be formulated as

5 $$\begin{aligned} \begin{aligned} \underset{S \subseteq {\mathcal {E}}_g}{\text {maximize}} \quad&T_{j \rightarrow i } \left( S \right) \\ \text {subject to} \quad&|S| \le k, \\&0 \le w_i \le w_{ub}, ~\sum ^k_{i = 1} w_i \le w_{max}, \text {where |.| denotes the cardinality of a set.} \end{aligned} \end{aligned}$$

#### Problem 2: rewiring problem

Given a weighted network $\mathcal {G_A}({\mathcal {V}}, \mathcal {E_A}, w_{{\mathcal {A}}})$, the problem is to maximize the information transfer between two given nodes by reconfiguring at most *k* existing edges. The modified network is given by $\mathcal {G_A} \cup {\mathcal {G}}_{\delta {\mathcal {A}}} = ({\mathcal {V}}, \mathcal {E_A} \cup {\mathcal {E}}_{\delta {\mathcal {A}} }, w_{{\mathcal {A}}} + w_{\delta {\mathcal {A}}} )$, where ${\mathcal {G}}_{\delta {\mathcal {A}}}$ denotes the modifications on the existing network. We require that the total weights of the modified edges be bounded by $w_{max}$ and each of the individual edge-weights $w^i_{\delta {\mathcal {A}}}$ be bounded by $w_{ub}$. The rewiring problem can be formulated as

6 $$\begin{aligned} \begin{aligned} \underset{{\mathcal {E}}_{\delta {\mathcal {A}}} \subseteq {\mathcal {E}}}{\text {maximize}} \quad&T_{j \rightarrow i } \left( {\mathcal {E}}_{\mathcal {A}} + {\mathcal {E}}_\mathcal {\delta A} \right) \\ \text {subject to} \quad&|{\mathcal {E}}_{\delta {\mathcal {A}}}| \le k, \\&0 \le w^i_{\delta {\mathcal {A}}} \le w_{ub}, ~\sum ^k_{i = 1} w^i_{\delta {\mathcal {A}}} \le w_{max}. \end{aligned} \end{aligned}$$

Below, we propose algorithms that solve Problems 1 and 2. First, we propose the algorithms for adding edges that maximize $T_{j\rightarrow i}$ in Problem 1. Next, to solve Problem 2, we propose an algorithm that removes edges with minimal contribution to the information transfer function. We then use the algorithms for Problem 1 to add new edges.

*Algorithms for Network Design (Problem 1):* We propose the Subgraph Completion Algorithm. This technique relies on the communicability centrality measure. From the definition of communicability in (4), shorter paths to node *j* contribute more to the communicability function. To increase the connectivity with shorter paths to node *j*, we form complete subgraphs between 2 nodes, with *j* as one of the two nodes, for all the possible combinations excluding node *i*. We then form complete subgraphs for all the combinations of $3,4,..(n-1)$ nodes, with *j* as one of the nodes. If further $|S| < k$, we arbitrarily add outgoing edges from node *i* to the rest of the nodes. We call this “Subgraph Completion Algorithm”, which is given in Algorithm 1 (Algorithms Section) and illustrated in Supplementary Fig. 4. We also use a Greedy Algorithm that computes the contribution of each edge towards $T_{j\rightarrow i}$ and selects the best edge whose contribution is the highest. The iteration continues until the added number of edges equals *k* (Supplementary note 5). Other commonly used algorithms include modular and complementary modular addition techniques (Methods-Algorithms).

*Algorithms for rewiring edges (Problem 2):* To maximize $T_{j\rightarrow i}$ for a given weighted network ${\mathcal {G}}_A\mathcal {(V,E_{A}}, w_{{\mathcal {A}}})$, associated with the system (1) by rewiring the topology, we remove the existing incoming edges to node *i* except from node *j* (Theorem 1, Supplementary Note 5). Let ${\mathcal {E}}_{i}$ denote this set of edges. If $|{\mathcal {E}}_{i}| \ge k$, we simply remove any *k* edges from ${\mathcal {E}}_{i}$. Else, if $|{\mathcal {E}}_{i}| \le k$, we look for other $k-|{\mathcal {E}}_{i}|$ edges to be removed. Towards this end, we introduce novel centrality measures that quantify (a) the causal inference of a node to the rest of the network (node-to-network influence) and (b) the effects (in terms of information transfer) received by a node from the network (network to node influence). Finally, we derive an *Information Transfer Edge Centrality* (*ITEC*) measure that quantifies the contributions of edges towards information transfers among nodes in the network. To define the *ITEC*, we first define the cause and the effect node centralities below.

***Cause centrality in complex network (node to network influence)***: Cause centrality, denoted by ${\mathcal {T}}_j$, quantifies the contribution of information/causal inferences by a node across the network. In other words, it quantifies the ability of a node *j* to transfer information across the network. For the system in (1) with adjacency matrix $A^T \in {\mathbb {R}}^{n\times n}$, the cause centrality of a node $j \in \{1,2\cdots n\}$ is given by

7 $$\begin{aligned} \begin{aligned} {\mathcal {T}}_j&= T_{j\rightarrow 1} + T_{j\rightarrow 2} + \cdots + T_{j\rightarrow n} = A_{1j}\frac{\sigma _{1j}}{\sigma _{11}} + A_{2j}\frac{\sigma _{2j}}{\sigma _{22}}+ \cdots + A_{nj}\frac{\sigma _{nj}}{\sigma _{nn}}\\&= A(:,j)^T*M(:,j); ~ \text {where}~~ M(i,j) = \frac{\Sigma (i,j)}{\Sigma (i,i)}. ~~ i,j \in \{1,2\cdots n\}. \end{aligned} \end{aligned}$$

***Effect centrality in a complex network (network to node influence)***: Effect centrality of a node *j*, ${\mathcal {R}}_j$ is defined as the amount of information received by a node *j* from all other nodes in the network. It measures the ability of nodes in a network to receive more “effects” or gather more information along the directed paths across the network. For the system in (1) with adjacency matrix $A^T \in {\mathbb {R}}^{n\times n}$, the effect centrality of a node $j \in \{1,2\cdots n\}$ is given by

8 $$\begin{aligned} \begin{aligned} {\mathcal {R}}_j&= A_{j1}\frac{\sigma _{j1}}{\sigma _{jj}} + A_{j2}\frac{\sigma _{j2}}{\sigma _{jj}}+ \cdots + A_{jn}\frac{\sigma _{jn}}{\sigma _{jj}} = A(j,:)*M(j,:)^T. \end{aligned} \end{aligned}$$

***Information Transfer Edge Centrality***: We combine the cause and effect centralities to derive a novel edge centrality measure based on information transfer. Intuitively, the contribution of an edge toward the information transfers across the network is related to the nodes it connects. If an edge connects a node with high cause centrality to a node with high effect centrality, then the edge has more influence on the information transfers across the network. Thus, the information transfer edge centrality of an edge (*i*, *j*), denoted as $ec_{ij}$ is $ec_{ij} = {\mathcal {T}}_i* {\mathcal {R}}_j,~ i,j \in \{1,2\cdots n\}.$

To remove $k-|{\mathcal {E}}_{i}|$ edges from the given network topology, we use the rankings provided by various edge centrality measures and remove the lowest rank *k* edges. We denote this set of edges to be removed by ${\mathcal {E}}_r$. We then use the Greedy Algorithm (Algorithm in Supplementary Note 5) or Subgraph Completion Algorithm (Algorithm 1, Methods) to add *k* new edges.

### Optimal assignment of edge weights

Let $S^*$ be the set of edges in the optimal topology which maximizes $T_{j\rightarrow i}$ with $|S^*| \le k$. We show that the optimal edge weights to be assigned lie on the boundary of the feasible weight set (Proposition 1 in Supplementary Note 5). Therefore, given the cardinality constraint *k*, optimal edge set $S^*$, $w_{max}$ and $w_{ub}$, compute $K_{ub} = \lfloor \frac{w_{max}}{w_{ub}}\rfloor$ and $K_{ubl} = w_{max}-K_{ub}w_{ub}$. Assign $w_{ub}$ to the first $K_{ub}$ elements in $S^*$. Assign $K_{ubl}$ to the next element in $S^*$ and 0 to the remaining edges.

### Approximation guarantee

Due to the NP-Hardness of our optimization problems, the solutions given by the algorithms are not guaranteed to be optimal. Finding an optimal solution requires the brute force method of finding all the *k* combinations of edges in the network and computing the information transfer. This method is intractable for moderate to large networks. We look at the structural set properties (submodular and supermodular) of our information transfer function to find an approximation guarantee of using the Greedy Algorithm in solving optimization problems. A set function, $f: 2^{{\mathcal {E}}}\rightarrow {\mathbb {R}}$ is called submodular if for all $P \subseteq Q \subseteq {\mathcal {E}}$ and $s\in {\mathcal {E}}\backslash Q$, it holds that $f(P \cup \{s\}) - f(P) \ge f(Q \cup \{s\}) - f(Q)$. If $-f$ is a sub-modular function, then *f* is called a super-modular function. Theorem 2 (Supplementary note 6) shows that the information transfer function is neither submodular nor supermodular. Therefore, the standard approximation guarantee^44^ provided by the Greedy Algorithm does not hold. Some recent works on optimizing set functions that are neither submodular nor supermodular show that the Greedy Algorithm can still provide performance guarantees. For example, in^45^, the authors employ the submodularity ratio, $\gamma$ and the curvature, $\alpha$ to define an approximation guarantee of greater than $\frac{1}{\alpha } (1-e^{-\alpha \gamma })f^*$ where $f^*$ denotes the optimal value. For a given non-negative set function *f*, the submodularity ratio is the largest $\gamma \in {\mathbb {R}}^+$ such that $\sum _{\omega \in \Omega \backslash S}\Delta _{\omega }(S) \ge \gamma \Delta _{\Omega }(S),~\forall ~{\Omega , S} \subseteq {\mathcal {E}}$. The curvature is the smallest $\alpha \in {\mathbb {R}}^+$ such that $\Delta _j(S\backslash j \cup \Omega ) \ge (1-\alpha ) \Delta _{j}(S\backslash j),~ \forall ~{\Omega , S} \subseteq {\mathcal {E}}, \forall {j} \in S\backslash \Omega$.

To justify the use of the Greedy Algorithm for solving the problems, we derive a positive lowerbound on $\gamma$ and an upperbound on $\alpha$ for our set function in the network topology defined by ${\mathcal {A}}_{0,1}$. In the ground set ${\mathcal {E}}_g$, the bounds on $\gamma$ and $\alpha$ are given by (Theorem 3, Supplementary note 5)

9 $$\begin{aligned} \gamma \ge \frac{T_{ji}(\omega _{ij})}{T_{ji}({\mathcal {E}}_g) - T_{ji}(\omega _{ij})}, \quad \alpha \le 1- \frac{T_{ji}(\omega _{ij})}{T_{ji}({\mathcal {E}}_g) - T_{ji}(\omega _{ij})}, \text {where}~~ \omega _{ij} = \{(j,i)\}, T_{ji} = T_{j\rightarrow i}. \end{aligned}$$

### Examples

**Figure 2:**
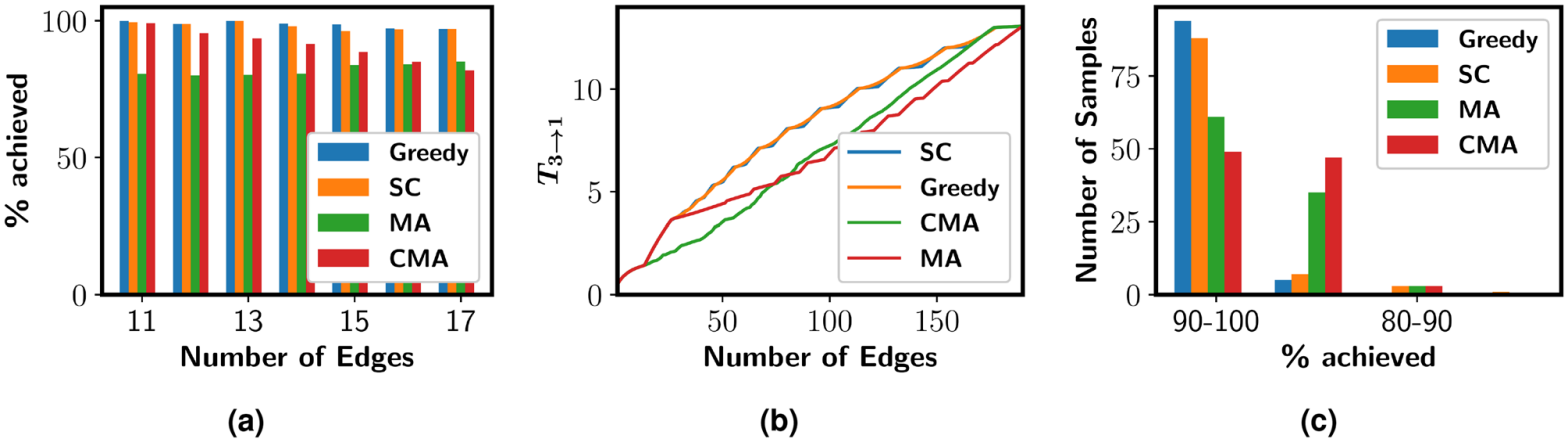
(**a**) Performance of different algorithms with respect to the optimum value for $n=6$, the input noise matrix $B= 0.1I_{6}$ and the initial covariance $\Sigma _0 =5 I_{6}$. The evolution of $T_{3\rightarrow i}$ is shown in Supplementary Fig. 5. (**b**) Performance of different algorithms for maximizing $T_{3\rightarrow 1}$ (**c**) Performances of different algorithms for maximizing $T_{5\rightarrow 2}$ for 100 random networks. We observe that out of the 100 random networks, the Greedy Algorithm achieves 90–100% of the optimum value for 94 networks, and for the rest of 6 networks, the greedy algorithm achieves 80–90% of the optimum value.

*Design Problem:* We first consider a small network of 6 nodes and analyze the performance of our heuristic algorithms for adding 11–17 edges that maximize $T_{3\rightarrow 1}$. We take the edge weights to be 1. To compare the results of our algorithms with the optimal value, we employ a brute force technique to find the optimal $T_{3\rightarrow 1}$ with 11–17 edges. Since the method requires an exhaustive search over different combinations, we restrict our analyses to 6 nodes. The performance comparison is shown in Fig. 2a. In all the figures, we denote the Subgraph Completion Algorithm by SC, the Greedy Algorithm by Greedy, Modular Addition, and Complementary Modular Addition by MA and CMA, respectively. We see that the Greedy Algorithm performs better than the rest, and the SC Algorithm performs closely to the Greedy Algorithm. Now, we look at the performance of the proposed algorithms at each stage of edge addition. Let the number of nodes be $n = 15$, and the objective is to maximize $T_{3\rightarrow 1}$. We take the input noise matrix $B= 0.1I_{15}$ and the initial covariance $\Sigma _0 = 5I_{15}$. After fixing the in-degree of node 1 and constructing the base topology, we have $n^2 - 2(n+1)$ = 197 possible edges. Out of these, 14 edges are self-loops. So, we need to select *k* out of 183 edges that maximize $T_{3 \rightarrow 1}$. The values of $T_{3\rightarrow 1}$ obtained for different values of *k* using the algorithms are shown in Fig. 2b. The constraint on the total weight is removed, and all the weights are assigned $w_{ub} = 1$.

*Update Problem:* In the update problem, we are a given network topology, and the goal is to add *k* edges that maximize $T_{j\rightarrow i}$. To compare the performance, we generate 100 randomly connected networks of 6 nodes and 10 edges. We use the above algorithms to add 5 new edges with $w_{max}=4.2$ and $w_{ub} = 1$ such that $T_{5\rightarrow 2}$ is maximized. Because of the complexity in finding the optimum value for $T_{5\rightarrow 2}$ (using the Brute force approach for comparison purposes) for large networks, we limit our analysis to a small network of 6 nodes. We take $\Sigma _0 = 5I_{6}$ and $B= 0.1I_{6}$. The performance comparison is shown in Fig. 2c.

*Computational Complexities:*The Greedy Algorithm is computationally expensive, bearing the worst-case computational complexity of ${\mathcal {O}}(n^4\beta _1 + n^4)$, where $\beta _1$ is the cost of computing the information transfer function and *n* is the number of edges to be added. The performance of the Subgraph Completion Algorithm is very close to the Greedy Algorithm with a significantly less computational complexity of ${\mathcal {O}}(n^4)$. A detailed comparison of the performances of these algorithms in terms of computational complexities and the maximization of the information transfers is given in Supplementary Note 6. An illustration of different topologies generated by the proposed algorithms for a network size of 20 nodes is also given.

*Approximation Guarantee:* From the definitions of submodularity ratio and curvature (Definitions, Supplementary Note 5), we compute $\gamma$ and $\alpha$ among all the subsets of ${\mathcal {E}}_g$ and select the largest and the smallest values respectively. We randomly generate 100 different subsets of *S* for a network of 50 nodes and determine the largest and smallest values of $\gamma$ and $\alpha$. The largest value of $\gamma$ has an average value of 0.9, signifying the closeness to submodularity empirically. The value of $\alpha$ ranges between 0 and 0.4, with an average value of 0.15. Thus using $\frac{1}{\alpha } (1-e^{-\alpha \gamma })f^*$, the Greedy Algorithm achieves over $80\%$, and it outperforms the worst-case approximation of $60\%$ for submodular functions.

## Applications to neurological networks

**Figure 3:**
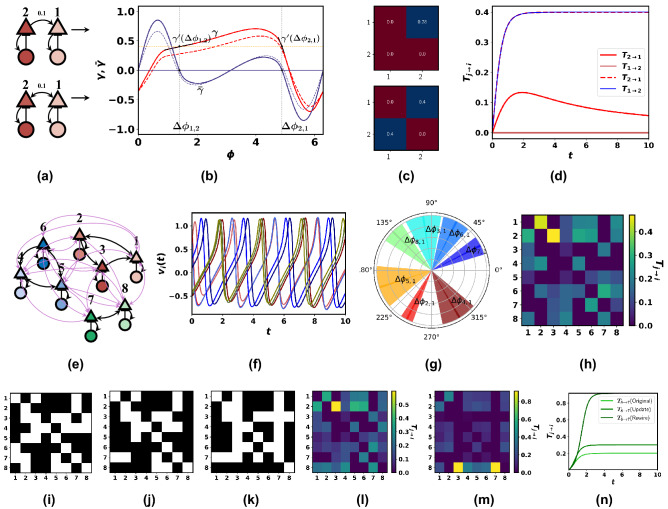
(**a**) The Wilson–Cowan neuronal oscillator consisting of two excitatory (triangle) and inhibitory (circle) neurons with average membrane potentials of *v* and *u* for two network topologies. The edge weight is 0.1. (**b**) The coupling function curves for both cases in figure (**a**). The dark red and blue curves show the coupling function and its antisymmetric curve for the bottom figure in (**a**). The other two dashed curves correspond to the coupling for the topology in the top Figure (**a**). (**c**) The transpose of coupling matrices found by linearizing the coupling functions shown in figure (**b**) around the zero crossings of $\bar{\gamma }$ in both cases. The (2, 1) element in the upper matrix is 0 as the corresponding network has no connection from 1 to 2 (**d**) $T_{j\rightarrow i}$ curves for both the topologies in Figure (**a**). The red curve shows $T_{2\rightarrow 1}$ for the upper topology. $T_{1\rightarrow 2}$ is 0 for this topology as there is no connection from node 1 to 2. The blue curve and the dotted red curve show $T_{1\rightarrow 2}$ and $T_{2\rightarrow 1}$ for the topology in bottom figure (**a**). As the coupling strengths are similar, the two information transfer curves overlap (**e**) Excitatory and inhibitory network of 8 nodes with couplings 0.015 and 0.1 (**f**) Oscillatory behaviours of the neurons (**g**) The phase differences fluctuate around a stable phase-locked state (darker lines) (**h**) Various information transfers among the excitatory neurons. (**i**) Given binary interconnection matrix of 8 nodes (**j**) Interconnection matrix using the update technique of adding 5 new edges with Greedy Algorithm (**k**) Interconnection matrix using the rewiring technique of 7 nodes using the Greedy Algorithm and *ITEC* centrality measure, (**l**) Information transfers among various excitatory neurons after updating with 5 edges (**m**) Information transfers among various excitatory neurons after rewiring using 7 edges, (**n**) Evolution of $T_{8\rightarrow 7}$ after the update and the rewiring process.

*Information flows in Neurological Networks:* We study the various information transfers among the excitatory populations of neurological networks. The dynamical interactions among the excitatory and inhibitory populations in a synaptically coupled neuronal network can be approximated by the Wilson–Cowan model of interacting oscillators (Supplementary note 8). In neurological networks, a single neuron fires repetitively when injected with a constant current. Therefore, it is reasonable to regard a simulated neuron as a limit cycle, at least for a certain small duration over a period of several spikes. We, therefore, assume that each oscillator *i* has an asymptotically stable periodic solution with frequency $\omega _i$. The couplings among the neurons are often only through weak input currents to the membrane potential of the cell. Thus, we assume weak couplings among the oscillators to prevent “Oscillator death”^46^. Moreover, when the couplings are weak, we can reduce the system of nonlinear equations to a set of equations on a torus using invariant manifold theory^46^. We then use averaging theory to obtain equations that depend only on the phase differences as (Supplementary Note 7)

10 $$\begin{aligned} d\phi _i = \bigg (\omega _i + \sum _j \gamma _{ij}(\phi _i-\phi _j)\bigg )dt + \sum \varsigma _{ik}dw_k \end{aligned}$$

where $\gamma _{i,j}$ denotes the coupling function between nodes *i* and *j* and the last term models external stochastic noise process, $\xi _i$ with covariance $\varsigma _i$, $w_k$ is a white noise Gaussian process with zero mean and unit covariance (Supplementary note 7). Because of the white noise process, strong deviations may occur that switches the dynamics to other stable states. When the noise levels are reduced, the expected time for such switching of the stable phase-locked states becomes arbitrarily large. In our work, we focus on finding the information transfer from a single dynamical state to another dynamical state. Therefore, we assume that the noise levels are small enough so that no such switching occurs during the relevant time intervals where the dynamical states communicate. The coupling function is computed by finding the response of the phase difference due to electrical synapse via gap junction potentials. A sensitivity analysis of the coupling function to noise levels, types of noise, and local noise is given in Supplementary Note 9. *Information transfer* between any two neurons in the network can be defined as an excitatory neuron’s influence on the excitation level of the second neuron and depends on the level of phase synchronization over the periodic interval. A popular and widely used theory in computing information transfers among neurons is that effective transmission of information between two oscillating neurons occurs when the pre-synaptic input of the sending neuron reaches the post-synaptic neuron at its maximum excitability phase, thereby amplifying the firing rate of the post-synaptic group. To compute the information transfers, we decompose the dynamics in (10) into a deterministic component and a fluctuating stochastic component. We estimate the stochastic component using linear approximations yielding a linear continuous stochastic model of the form in (1) (Methods and Supplementary Note 8). We show that changes in the network topology alter the information transfers among neurons and that by designing the correct topology, we can control the information transfers to modify undesired excitation levels or achieve desired patterns of information transfers. Change in network topology can be due to endogenous changes promoting physiological or pathological conditions or exogenous interventions. We assume the initial state covariance of the fluctuating components is $0.1I_8$, and the input noise matrix is taken as $0.001I_8$. We first show in Fig. 3a–d that a change in the interactions among the neurons induces a change in the stable phase-locked states and eventually in the coupling strength and information transfers. Next, we show in Fig. 3h–n how we can use our proposed algorithms in the previous section to maximize $T_{8\rightarrow 7}$ for the network shown in Fig. 3e. Figure 3f illustrates the oscillatory dynamics of the neurons and in Figure 3g, we demonstrate the variations of the phase difference around a stable point.

*Update Problem:* We consider the given neural network in Fig. 3e for both the update and rewiring problems. We take the initial state covariance for the states $\phi _i$ as $0.1I_8$ and the input noise matrix as $0.001I_8$. The adjacency matrix has entries given by $G_{i,k}$. Note that $G_{i,j}$ depends on the phase difference and the phase response curve. Also, $G_{i,j} = 0$ if there is no edge from *i* to *j* (see Methods). The update problem is to add 5 edges such that $T_{8 \rightarrow 7}$ is maximized. The upper bounds on the weights are given by $w_{max} = 0.07, w_{ub} = 0.015$. Note that the coupling matrix given in Fig. 3c should not be confused with the weights $w_{max}$ and $w_{ub}$. The edge weights are denoted by the black (0.1) and purple arrows (0.015) in the network in Fig. 3e.

*Rewiring Problem:* We continue with the example of the neural network in Fig. 3e for the rewiring problem to maximize $T_{8\rightarrow {7}}$. We restrict the number of edges that can be reconfigured to 7. Following Algorithm 2, we first remove the three edges sinking in node 7 (excluding the edge from node 8). The remaining 4 edges to be removed are found from the lowest rank edge rankings based on the *ITEC*. The bounds on the weight are given by $w_{max} = 0.1$ and $w_{ub} = 0.015$.

These results validate the postulation that the functional information transfers among the neurons depend on the underlying network topology, which may occasionally change due to physiological or pathological conditions.

## Discussion

This report provides a generic mechanism to quantify the information transfers among nodes in complex network systems. For a network system with linear stochastic dynamics, we define information transfer as the difference between the marginal entropies. For weakly coupled oscillators with stochastic fluctuations, we show that the information transfer is a function of the state covariance and the coupling strengths among the oscillators. We show that the formulation is consistent with Schreiber’s transfer entropy and Horowitz’s thermodynamical information flow (Supplementary node 3). We provide supporting examples that indicate the change in information transfer patterns because of network topology changes. For networks of weakly coupled oscillators, the theory is based on a linear approximation of the phase dynamics around the stable phase-locked states. The method thus highlights the significance of phase synchronizations in the study of weakly coupled oscillators.

The structural analysis of the information transfer function reveals that the information transfer is a monotone-increasing function under specific conditions. The $NP-hardness$ of the function forces us to define an approximation guarantee when using the Greedy algorithm. Also, the information transfer function is proven to be neither a submodular nor a supermodular function. These conditions place the context of our study outside the standard submodular or supermodular functions, preventing the use of the standard approximation guarantee of $(1-1/e) \approx 63.21\%$ (of the optimal value for submodular functions). However, these conditions are favourable because the complexities are reduced by minimizing the search space to only those edges with positive contributions. Also, we show that the information transfer function enjoys an approximation guarantee of more than $80\%$ when we use the Greedy Algorithm. For assigning the edge weights, we proved that optimal edge weights to be assigned to the set of new edges lie on the boundary of the feasible weight set.

Information transfer, in the context of neurological networks, is defined by the amount of influence of one node on the excitation levels of a neighbouring node and depends on the level of phase synchronization. We computed the various information transfers among the neurons in a Wilson–Cowan model of 8 neurons. Finally, using the proposed algorithms, we maximized information transfer between two prespecified excitatory neurons. While the theory in this report focuses on maximizing information transfers by finding the near-optimal topology, there are other possible scopes that we can explore to control information transfer. For example, if the system in (1) is controllable with an input matrix defining the controllable nodes in the network, then we can study the variations in information transfer due to varying inputs. Hybrid control of the topology (passive) and external control (active) may provide more flexibility in controlling information transfer.

## Methods

### Algorithms

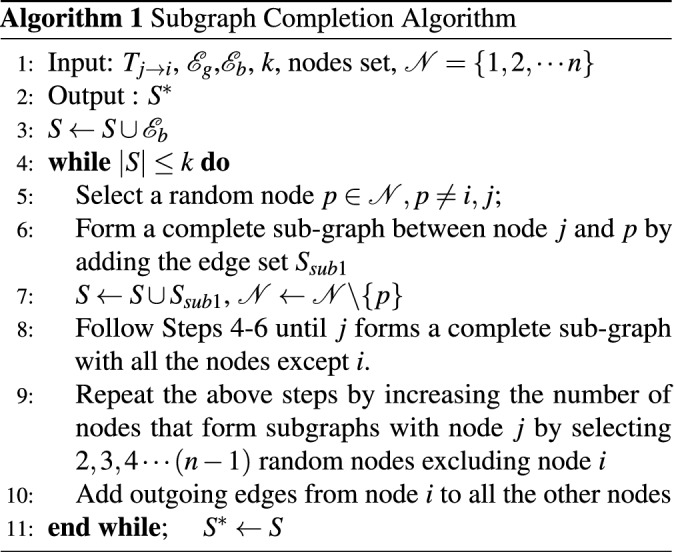

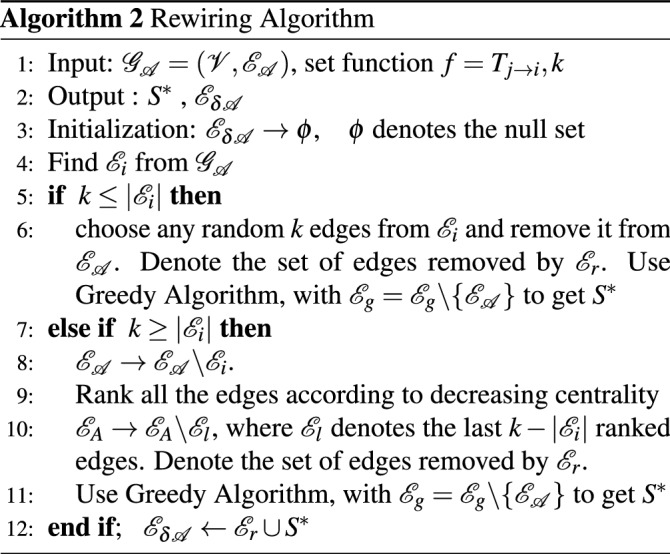





Modular Addition Technique: ^47^ In this approach, we compute $T_{j\rightarrow i}$ for each potential edge in the network. The edges are then sorted in decreasing order of their contribution to $T_{j\rightarrow i}$. The first *k* edges are then used for maximizing $T_{j\rightarrow i}$.

***Complementary Modular Addition Technique:***^47^ Given the ground set, ${\mathcal {E}}_g$ , we compute $f({\mathcal {E}}_g) - f({\mathcal {E}}_g)\backslash {i}, \forall {i} \in {\mathcal {E}}_g$ where *f* is $T_{j\rightarrow i}$. The edges are then sorted in descending order and the first *k* links are added to the base topology.

### Reducing the phase dynamics into linear stochastic dynamics

We assume that in the unperturbed system ($\varsigma _{ik} = 0$), the phase dynamics in (10) has a stable phase-locked state with a constant phase difference, $\Delta \phi _{ij} = \phi _i^{ref}- \phi _j^{ref}$ and a collective oscillation frequency, $\Omega$, that is for all $i \in \{1, \cdots N\}$, $\Omega = \omega _i + \sum _j \gamma _{ij}(\Delta \phi _{i,j})$. We decompose the phase dynamics into a deterministic reference part, $\phi _i^{ref}$, and a fluctuating part, $\phi _i^{fluc}$. The solution to the deterministic dynamics is given by $\phi _i^{ref}(t) = \Omega t + \Delta \phi _{i,1}^{ref}$. Introducing new coordinates, $\varphi _i = \phi _i-\phi _i^{ref}$, (10) can be written as $d\varphi = f(\varphi )dt + \varsigma dw$, where $f_i(\varsigma ) = \omega _i + \sum _j\gamma _{ij}(\varsigma _i-\varsigma _j+\Delta \phi _{i,j}^{ref})- \Omega$. We assume that the noise levels, $\varsigma _{ik}$ are small and linearizing around the stable phase-locked states, we get a linear continuous stochastic model as

$$\begin{aligned} d\varphi = G\varphi dt + \varsigma dw,\quad \text {where} \quad G_{ij} = \gamma _{ij}'\left(\Delta \phi _{i,j}^{ref}\right). \end{aligned}$$

## Supplementary Information

Below is the link to the electronic supplementary material.

Supplementary Information.

## Data Availability

The codes/data used during the current study are available from the corresponding author upon reasonable request.
