# Supplementary material for: On quantification and maximization of information transfer in network dynamical systems
Source: Sci Rep. 2023 Apr 5;13:5588. doi: 10.1038/s41598-023-32762-7 (PMC10076297; doi:10.1038/s41598-023-32762-7)
Supplement: Supplementary file 1 — Supplementary Information. [file 41598_2023_32762_MOESM1_ESM.pdf]

Supplementary Figure 1

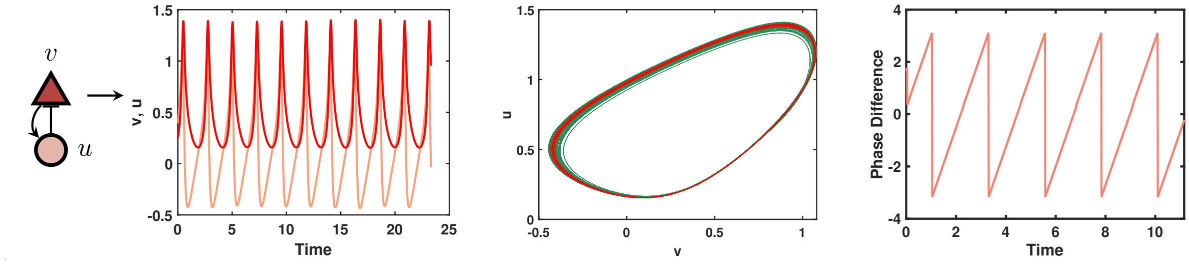

(a) (b) (c) (d)

Figure 1: (a) The Wilson-Cowan neuronal oscillator consisting of a single excitatory (triangle) and inhibitory (circle) neurons with average membrane potentials of v and u (b) Oscillatory activities of excitatory and inhibitory population in (a), (c) The Phase plane dynamics. Phase portrait shows the stochastic trajectories of (b) (green) together with the deterministic limit cycle (red) (d) Phase signal obtained from mapping the stochastic trajectory in (b) onto the phase field

Supplementary Figure 2

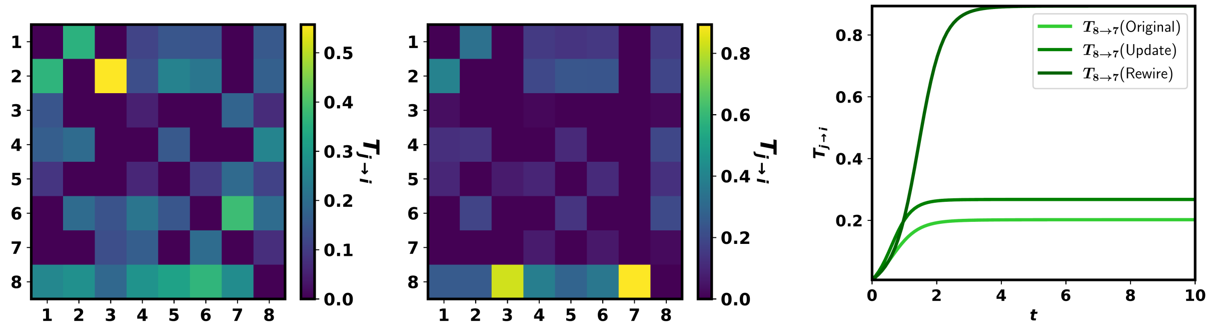

(a) (b) (c)

Figure 2: (a) Information transfers among neurons due to addition 5 new edges using the Subgraph Completion Algorithm in the original network given in Fig. 3e of the main paper (b) Information transfers among neurons due to rewiring 7 new edges and using the Subgraph Completion Algorithm in the Original network given in Fig.3e of the main paper (c) Information transfer from excitatory neuron 8 to 7. $T_{8\to 7}$ is maximized with the rewiring algorithm. The performance is almost similar to using Greedy Algorithm (as shown in Figure 3(i-m) of the main paper)

Supplementary Figure 3

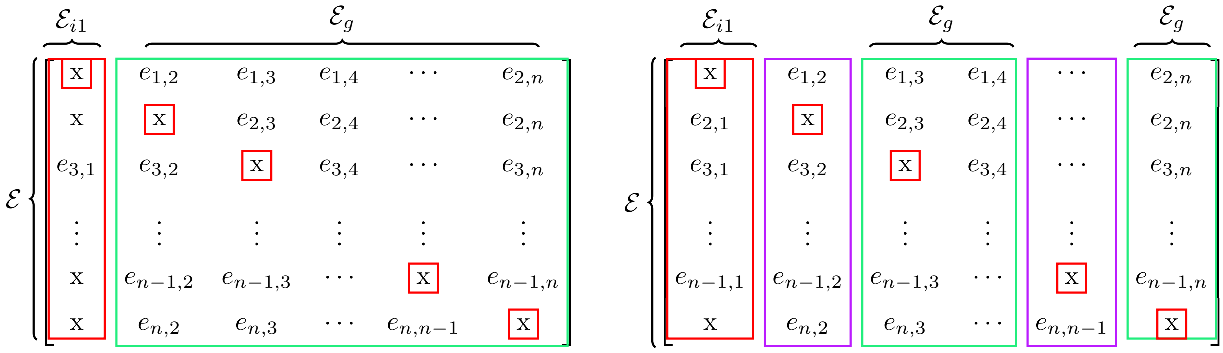

Figure 3: The left panel shows the case when there is a single input to node $i = 1$ from node $j = 3$. To maximize $T_{3\to1}$ by adding edges, we pick edges from the set $\mathcal{E}_g$, defined by the green box. We restrict any new incoming edges to node i, defined by the red box. We also do not consider the self-loops denoted by the diagonal elements. The right panel shows the case with additional edges to node 1 from nodes 2 and $n-1$. The incoming edges to node 2 and $n-1$ inside the purple boxes are considered only after all the edges in $\mathcal{E}_g$ are added.

Supplementary Figure 4

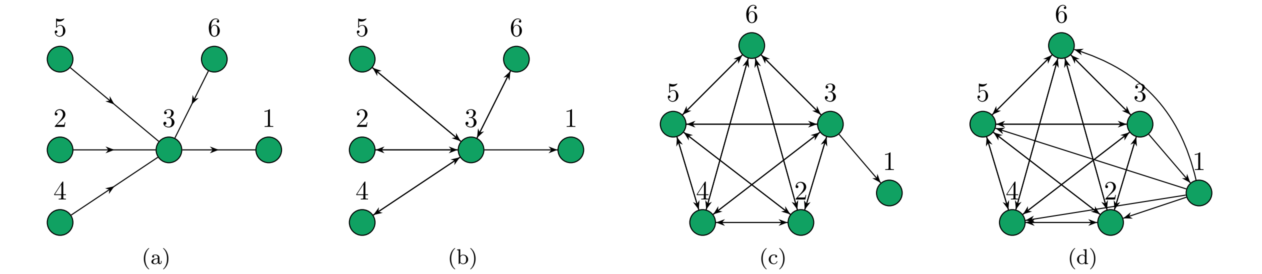

Figure 4: Illustration of Subgraph-Completion Algorithm for maximizing Information transfer from node 3 to node 1. Fig. (a) shows the connected network topology with minimum edges that maximizes $T_{3\to1}$. We call this the 'Base Topology' in the main manuscript.

Supplementary Figure 5

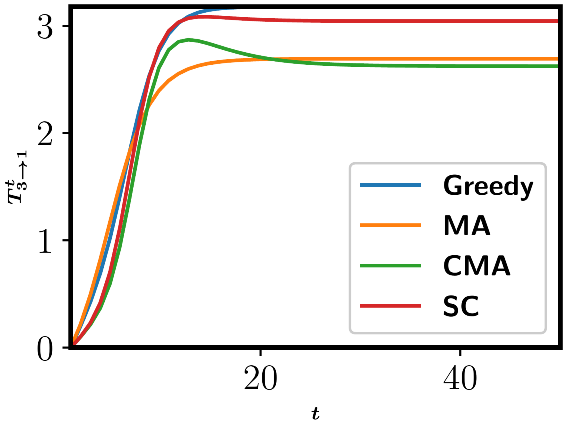

Figure 5: Performance of different algorithms for maximizing $T_{3\to1}$ in four different networks with 16 edges, $w_{ub} = 1$ and $w_{max} = 15.5$ for the design problem in Figure 2a of the main paper.

Supplementary Figure 6

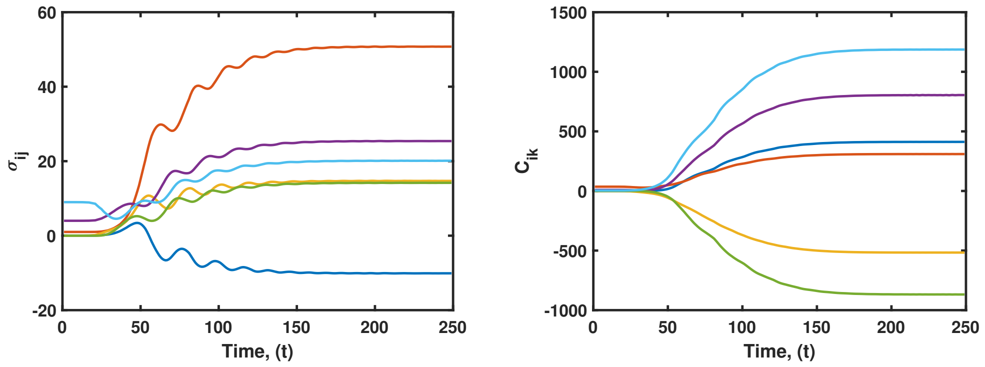

(a) (b)

Figure 6: (a) Evolution of the elements of the state covariance matrix (b) Evolution of the elements of the cofactor of the state covariance matrix

Supplementary Figure 7

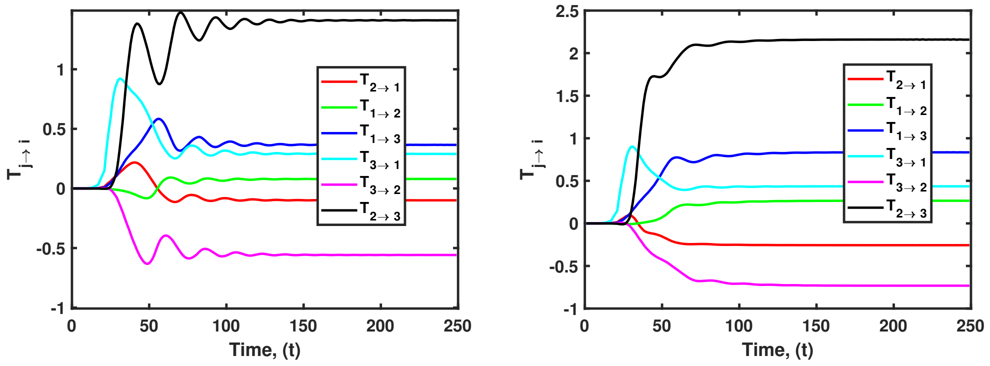

(a) (b)

Figure 7: (a) The Liang-Kleeman information transfers (b) Horowitz-Esposito information flows

Supplementary Figure 8

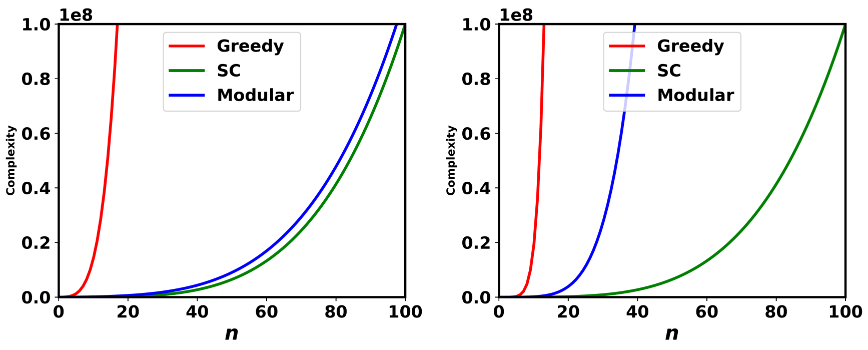

(a) (b)

Figure 8: (a) Complexities of the algorithms for a network of 10 nodes and n edges (b) Complexities of the algorithms for a network of arbitrary nodes and n edges

Supplementary Figure 9

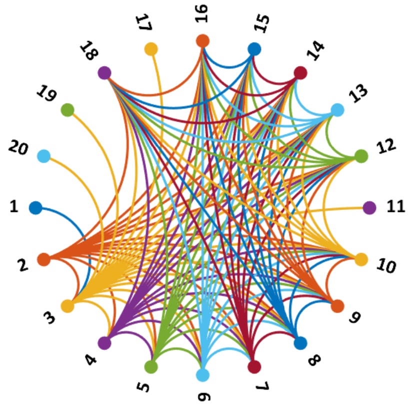

(a)

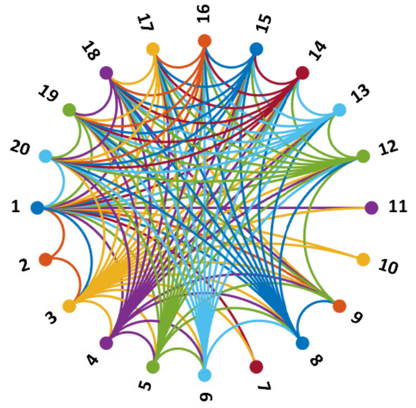

(b)

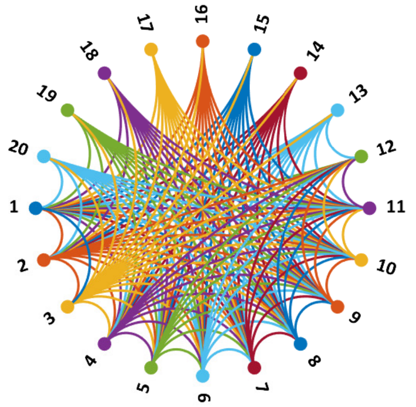

(c)

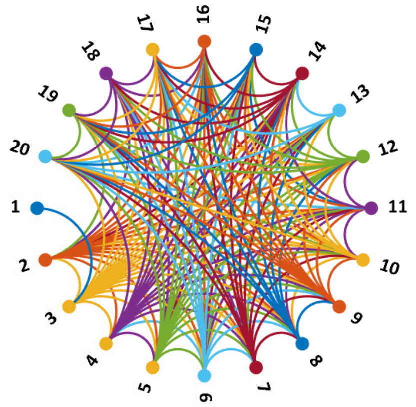

(d)

Figure 9: Network topologies for maximizing $T_{3\to1}$ by adding 200 edges using (a)Greedy Algorithm (b) Subgraph-Completion Algorithm (c) Modular Approach (d) Complementary Modular Approach

Supplementary Figure 10

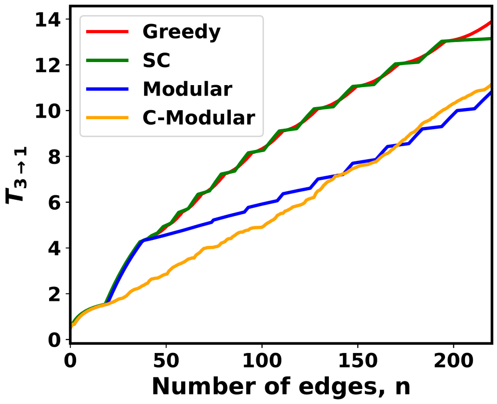

(a)

Figure 10: Performance comparisons for maximizing $T_{3\to1}$ by adding 220 new edges in a network of 20 nodes

Supplementary Figure 11

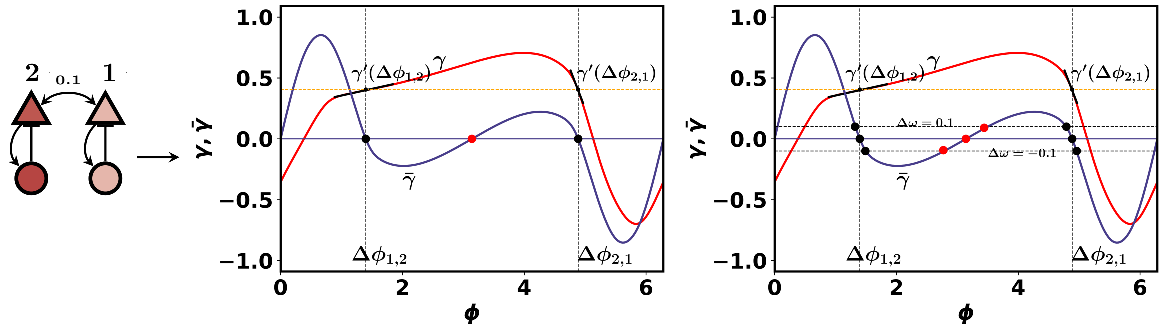

(a) (b) (c)

Figure 11: (a) The Wilson-Cowan neuronal oscillator consisting of two excitatory (triangle) and inhibitory (circle) neurons with average membrane potentials of v and u. (b) The coupling function curves for the network in figure (a). The dark red and blue curves show the coupling function and its antisymmetric curve. The black dots denote the stable synchronous and anti-phase states, the orange dot denotes the unstable non-synchronous solution (c) Coupling function with varying heterogeneity

Supplementary Figure 12

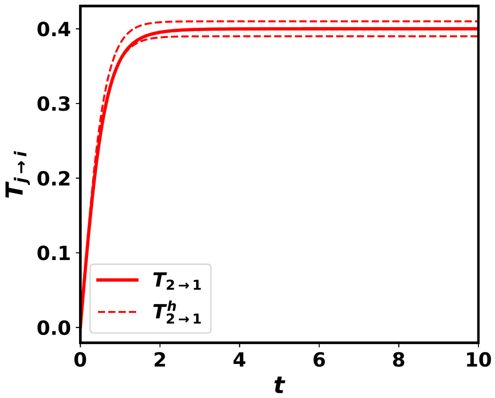

Figure 12: Variations in $T_{2\to1}$ due to weak heterogeneity, denoted by $T^h_{2\to1}$

Supplementary Figure 13

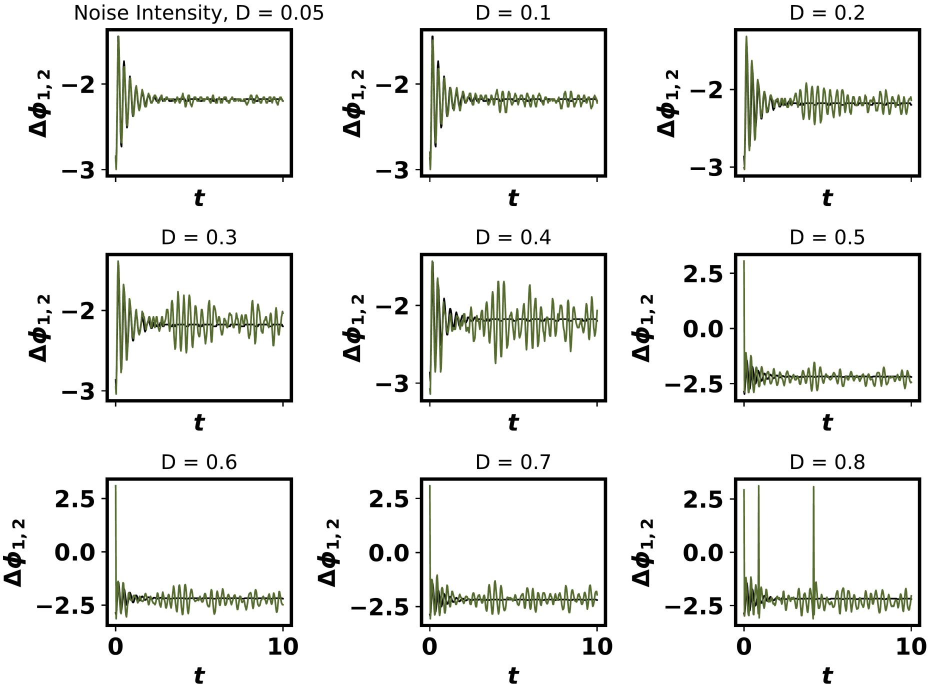

Figure 13: Variations in $\Delta\phi_{1,2}$ due to the effect of various intensities of additive noise

Supplementary Figure 14

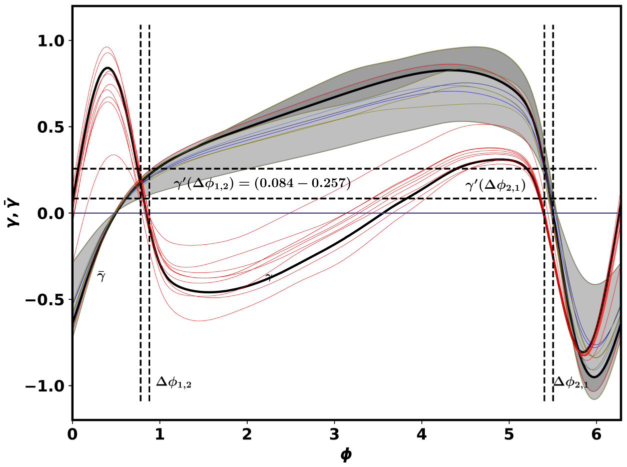

Figure 14: Variations in $\gamma, \bar{\gamma}$ due to the effect of various intensities of additive noise. The black curves are the coupling functions in the noiseless case. The grey area denotes the variations in the coupling function. For small noise intensity, D = 0.05, the variations in $\Delta\phi_{1,2}$ and $\gamma_{1,2}$ are relatively small.

Supplementary Figure 15

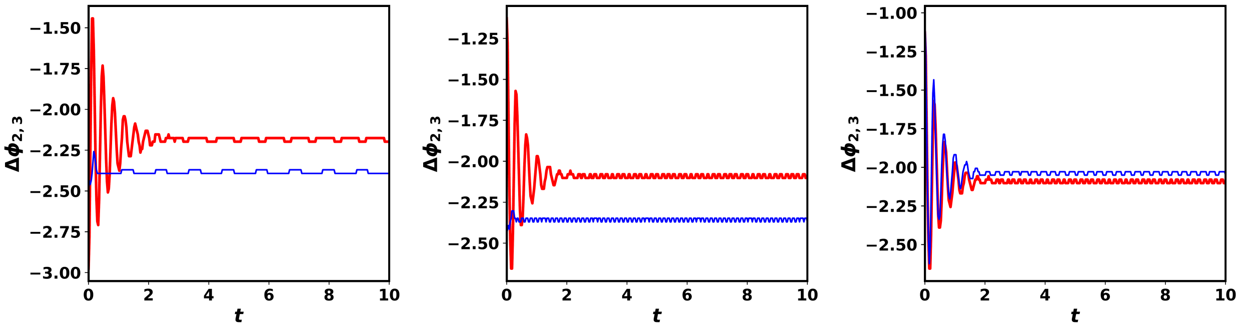

(a) (b) (c)

Figure 15: The red curves denote the evolution of phase differences when there is no multiplicative noise. The blue curves denote the evolution under the influence of multiplicative noise of intensity, D= 0.02 (a) A change in the synchronization pattern under the influence of multiplicative noise (b) Effect of multiplicative noise on the phase difference of two uncoupled oscillators (c) Effect of multiplicative noise on stable phase-locked states

Supplementary Figure 16

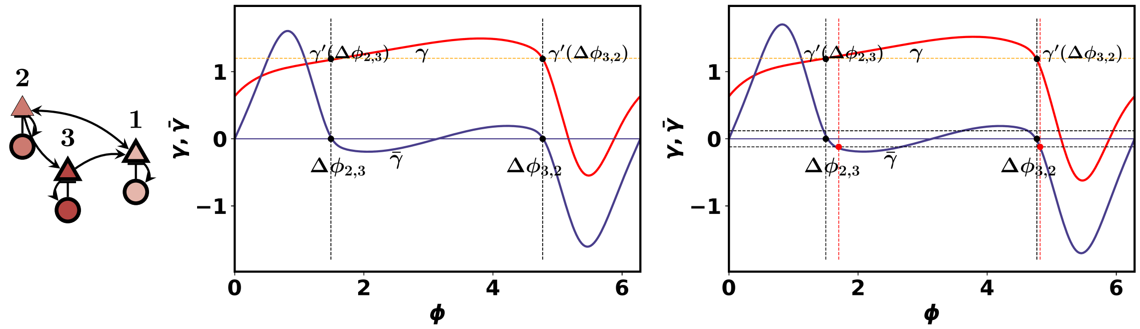

(a) (b) (c)

Figure 16: (a) The Wilson-Cowan neuronal oscillator consisting of three excitatory (triangle) and inhibitory (circle) neurons with average membrane potentials of v and u. (b) The coupling function curve, $\gamma_{2,3}$ for the network in figure (a). The dark red and blue curves show the coupling function and its antisymmetric curve. The black dots denote the stable synchronous and anti-phase states (c) Coupling function with varying local noise

Supplementary Figure 17

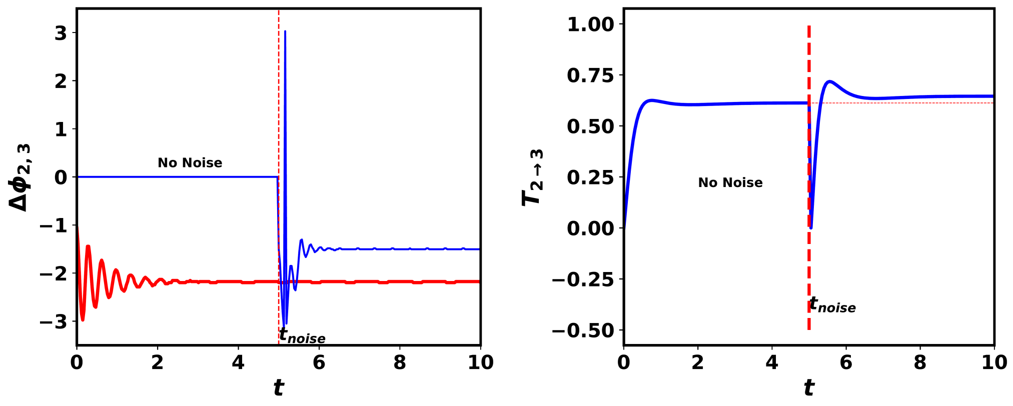

(a) (b)

Figure 17: (a) Shift in the stable phase-locked state due to the multiplicative noise(b) $T_{2\to3}(t)$ increases from 0.613 to 0.654 due to the multiplicative noise induced at $t = 5$ secs.

Supplementary Note 1
Information Transfer in Dynamical Systems

The formulation of information transfer in [1–3] is based on freezing a direction of the phase space and is derived for a general stochastic system using the Fokker-Planck equation. This section defines the information transfer between any two states for a general dynamical system. Consider a dynamical system in $\mathbb{R}^2$ with states x_1 and x_2 drawn from a probability distribution $\rho(\boldsymbol{x})$, $\boldsymbol{x} = (x_1, x_2)^T$ and vector field, $F = (F_1, F_2)'$, defined as

$$\frac{dx_1}{dt} = F_1(x_1, x_2, t), \quad \frac{dx_2}{dt} = F_2(x_1, x_2, t). \tag{1}$$

Correspondingly, we have the differential entropy, H associated with $\rho(\boldsymbol{x})$ defined as $H = -\int \rho(\boldsymbol{x}) \ln \rho(\boldsymbol{x}) d\boldsymbol{x}$. The marginal entropies are given by $H_i = -\int \rho_i \ln \rho_i dx_i, \; i = 1, 2$, where ρ_i denotes the marginal distribution of state x_i. As the system evolves in time, $\frac{dH_1}{dt}$ is contributed from two sources, one from x_1 alone and the remaining is the contribution from the state x_2. The latter can be interpreted as the amount of information transfer from x_2 to x_1. In terms of cause-effect relation, the latter is the amount of cause from x_2 that affects the evolution of x_1. Denoting the former as $\frac{dH_1^*}{dt}$ and the latter as $T_{2\to1}$, we can thus write

$$\frac{dH_1}{dt} = \frac{dH_1^*}{dt} + T_{2\to1} \tag{2}$$

For a system in $\mathbb{R}^n$, (2) can be written as [1–3]

$$T^t_{j\to i} = \frac{dH_i}{dt} - \frac{dH_{i\not{j}}}{dt} \tag{3}$$

Similar to (2), $\frac{dH_{i\not{j}}}{dt}$ in the above equation can be interpreted as the rate of change of marginal entropy of x_i with contributions from all other states except from x_j. To compute $\frac{dH_{i\not{j}}}{dt}$, we freeze the evolution of state x_j in the phase space and compute the marginal entropy of x_i.

Now, consider the stochastic system below with states x_1 and x_2 drawn from a probability distribution $\rho(\boldsymbol{x})$, $\boldsymbol{x} = (x_1, x_2)^T$ and vector field, $F = (F_1, F_2)'$,

$$dx_i(t) = F_i(x_1, x_2, t)dt + G_i(x_1, x_2, t)dW_i(t), \quad i = 1, 2$$

The information transfer from x_2 to x_1 is given by

$$T_{2\to1} = \frac{dH_1}{dt} - \frac{dH_{1\not{2}}}{dt} \tag{4}$$

The density evolution is given by the Fokker-Planck equation

$$\frac{\partial \rho(\boldsymbol{x})}{\partial t} + \frac{\partial}{\partial x_1}(F_1\rho(\boldsymbol{x})) + \frac{\partial}{\partial x_2}(F_2\rho(\boldsymbol{x})) = \frac{1}{2}\sum_{i,j=1}^{2} \frac{\partial^2 (g_{11}\rho_{1\not{2}})}{\partial x_i \partial x_j} \tag{5}$$

where $g_{ij} = g_{ji} = \sum_{k=1}^{2} G_{ik}G_{jk}$. Integrating eq. (5) with respect to x_2 gives the evolution of ρ_1:

$$\frac{\partial \rho_1}{\partial t} + \int \frac{\partial}{\partial x_1}(F_1\rho))dx_2 = \frac{1}{2}\int \frac{\partial^2 (g_{11}\rho)}{dx_1^2} dx_2 \tag{6}$$

Multiplying eq. (6) by $-(1 + log\rho_1)$ and followed by integration with respect to x_1, we get

$$\frac{dH_1}{dt} - \int\int log\rho_1 \frac{\partial (F_1\rho)}{\partial x_1} dx_1 dx_2 = -\frac{1}{2}\int\int log\rho_1 \frac{\partial^2 (g_{11}\rho)}{\partial x_1^2} dx_1 dx_2 \tag{7}$$

Integrating by parts, eq. (7) reduces to

$$\frac{dH_1}{dt} = -E\left(F_1 \frac{\partial log\rho_1}{\partial x_1}\right) - \frac{1}{2}E\left(g_{11}\frac{\partial^2 log\rho_1}{\partial x_1^2}\right) \tag{8}$$

where E is the expectation. To arrive at the formulation of $T_{2\to1}$ from eq. (4), we need to evaluate $H_{1\not{2}}$. Over the small time interval, $[t, t + \Delta t]$, $H_{1\not{2}}(t + \Delta t)$ is the marginal entropy of x_1 at time $t + \Delta t$ as x_2 is frozen as a parameter instantaneously at t. Clearly, we cannot use the Fokker-Planck equation to derive $H_{1\not{2}}$ since the dynamics are inconsistent through the time duration. We have

$$\frac{dH_{1\not{2}}}{dt} = lim \frac{H_{1\not{2}}(t + \Delta t) - H_1(t)}{\Delta t}$$

If we denote $x_{1\not{2}}$ as a parameter in the interval $[t, t + \Delta t]$, we have

$$dx_{1\not{2}} = F_1(x_{1\not{2}}, x_2, t) + \sum_k G_{1k} dw_k \tag{9}$$

Correspondingly, the density $\rho_{1\not{2}}$ evolves following the Fokker-Planck equation

$$\frac{\partial \rho_{1\not{2}}}{\partial t} + \frac{\partial}{\partial x_1}(F_1 \rho_{1\not{2}}) = \frac{1}{2}\frac{\partial^2 (g_{11}\rho_{1\not{2}})}{\partial x_1^2}, \quad t \in [t, t + \Delta t] \tag{10}$$

Introduce the function $f_t(x_1) = log\rho_{1\not{2}}(t, x_1)$, whose evolution is obtained by dividing eq. (10) by $\rho_{1\not{2}}$:

$$\frac{\partial f_t}{\partial t} + \frac{1}{\rho_{1\not{2}}}\frac{\partial}{\partial x_1}(F_1 \rho_{1\not{2}}) = \frac{1}{2\rho_{1\not{2}}}\frac{\partial^2 (g_{11}\rho_{1\not{2}})}{\partial x_1^2}, \quad t \in [t, t + \Delta t] \tag{11}$$

Discretizing eq. (11), we have

$$f_{t+\Delta t}(x_1) = f_t(x_1) - \frac{\Delta t}{\rho_{1\not{2}}}\frac{\partial}{\partial x_1}(F_1 \rho_{1\not{2}}) + \frac{\Delta t}{2\rho_{1\not{2}}}\frac{\partial^2 (g_{11}\rho_{1\not{2}})}{\partial x_1^2}, \quad t \in [t, t + \Delta t] \tag{12}$$

For the 2D case, we have $\rho_{1\not{2}} = \rho_1$, therefore, eq. (12) can be written as

$$f_{t+\Delta t}(x_{1\not{2}}(t+\Delta t)) = f_t(x_{1\not{2}}(t+\Delta t)) - \frac{\Delta t}{\rho_1}\frac{\partial}{\partial x_1}(F_1 \rho_{1\not{2}}) + \frac{\Delta t}{2\rho_1}\frac{\partial^2 (g_{11}\rho_1)}{\partial x_1^2}, \quad t \in [t, t + \Delta t] \tag{13}$$

The term $x_{1\not{2}}(t + \Delta t)$ can be expanded as

$$x_{1\not{2}}(t + \Delta t) = x_1(t) + F_1 \Delta t + \sum_k G_{1k}\Delta w_k + \text{higher order terms} \tag{14}$$

Substituting back in eq. (13) and performing Taylor series expansion, we get

$$\begin{aligned} f_{t+\Delta t}(x_{1\not{2}}(t+\Delta t)) &= f_t(x_1 + F_1\Delta t + \sum_k G_{1k}\Delta w_k)) - \frac{\Delta t}{\rho_{1\not{2}}}\frac{\partial}{\partial x_1}(F_1\rho_{1\not{2}}) + \frac{\Delta t}{2\rho_1}\frac{\partial^2(g_{11}\rho_1)}{\partial x_1^2} \\ &= f_t(x_1) + \frac{\partial f_t}{\partial x_1}\left(F_1\Delta t + \sum_k G_{1k}\Delta w_k\right) + \frac{1}{2}\frac{\partial^2 f_t}{\partial x_1^2}(F_1\Delta t + \sum_k G_{1k}\Delta w_k)^2 - \frac{\Delta t}{\rho_1}\frac{\partial (F_1\rho_1)}{\partial x_1} + \\ &\qquad \frac{\Delta t}{2\rho_1}\frac{\partial^2(g_{11}\rho_1)}{\partial x_1^2} \end{aligned} \tag{15}$$

Taking expectations on both sides, the left-hand side is $-H_{1\not{2}}(t + \Delta t)$, and the first term on the right-hand side is $-H_1(t)$. The second term on the right-hand side is given by

$$\Delta t E\left(F_1 \frac{\partial f_t}{\partial x_1}\right) + E\left(\frac{\partial f_t}{\partial x_1}\sum_k G_{1k}\Delta w_k\right) = \Delta t E\left(F_1\frac{\partial f_t}{\partial x_1}\right) \tag{16}$$

The third term after expansion can be written as

$$\frac{1}{2}E\left[\frac{\partial^2 f_t}{\partial x_1^2}\sum_k G_{1k}\Delta w_k \sum_j G_{1j}\Delta w_j\right] = \frac{1}{2}E\left[\frac{\partial^2 f_t}{\partial x_1^2}\sum_k G_{1k}^2(\Delta w_k)^2 + \sum_{k\neq j} G_{1k}G_{1j}\Delta w_k \Delta w_j\right] \tag{17}$$

Summation over $k \neq j$ vanishes after the expectation is performed. The first summation is equal to $g_{11}\Delta t$. Putting all these together in eq. (113), we get

$$H_{1\not{2}}(t+\Delta t) = H_1(t) - \Delta t E\left(F_1\frac{\partial log\rho_1}{\partial x_1}\right) - \frac{\Delta t}{2}E\left(g_{11}\frac{\partial^2 log\rho_1}{\partial x_1^2}\right) + \Delta t E\left(\frac{1}{\rho_1}\frac{(\partial F_1\rho_1)}{\partial x_1}\right) - \frac{\Delta t}{2}E\left(\frac{1}{\rho_1}\frac{\partial^2(g_{11}\rho_1)}{\partial x_1^2}\right) \tag{18}$$

The second and fourth terms on the right-hand side can be combined to give $\Delta t E(\frac{\partial F_1}{\partial x_1})$. So,

$$\frac{dH_{1\not{2}}}{dt} = E\left(\frac{\partial F_1}{\partial x_1}\right) - \frac{1}{2}E\left(g_{11}\frac{\partial^2 log\rho_1}{\partial x_1^2}\right) - \frac{1}{2}E\left(\frac{1}{\rho_1}\frac{\partial^2(g_{11}\rho_1)}{\partial x_1^2}\right) \tag{19}$$

The second and the third terms on the right-hand side of eq. (19) are from the stochastic perturbations. Using eq. (8) and eq. (19) in eq. (4), information transfer from x_2 to x_1 is given by

$$T_{2\to 1} = -E\left(\frac{1}{\rho_1}\frac{\partial(F_1\rho_1)}{\partial x_1}\right) + \frac{1}{2}E\left(\frac{1}{\rho_1}\frac{\partial^2(g_{11}\rho_1)}{\partial x_1^2}\right) \tag{20}$$

The first term in eq. (20) is because of the deterministic system and the second term is the contribution from stochasticity. If g_{11} is independent of x_2, the second term vanishes. That is

$$E\bigg(\frac{1}{\rho_1}\frac{\partial^2(g_{11}\rho_1)}{\partial x_1^2}\bigg) = \int \frac{\partial^2(g_{11}\rho_1)}{\partial x_1^2}dx_1 = 0 \tag{21}$$

Thus, for stochastic systems with noise input matrix independent of the states, the information transfer is given as

$$T_{2\to 1} = -E\bigg(\frac{1}{\rho_1}\frac{\partial(F_1\rho_1)}{\partial x_1}\bigg) = -\int\int \rho_{2|1}(x_2|x_1)\frac{\partial F_1\rho_1}{\partial x_1}dx_1dx_2 \tag{22}$$

Combining eq. (8), (19) and (22), for the 2D system, we have

$$\frac{dH_1}{dt} + \frac{dH_2}{dt} = \frac{dH}{dt} + T_{1\to 2} + T_{2\to 1} \tag{23}$$

From eq. (23), we infer that unlike the flow of matter, the information does not necessarily have to be lost in a component for the other to receive it. Similar to eq. (22), we can show that for a general dynamical system in $\mathbb{R}^n$ with states $\boldsymbol{x} = (x_1, x_2, \cdots x_i, \cdots x_j, \cdots x_n)^T$,

$$T^t_{j\to i} = -E\bigg[\frac{1}{\rho_i}\int_{\mathbb{R}^{n-2}} \frac{\partial F_i\rho_{\not j}}{\partial x_i}\bigg] = -\int_{\mathbb{R}^n} \rho_{j|i}\frac{\partial \rho_{\not j}F_i}{\partial x_i}d\boldsymbol{x} \tag{24}$$

where $\rho_{\not j}$ denotes the joint distribution of $(x_1, \cdots x_{j-1}, x_{j+1}, \cdots x_n)$ at time t.

Supplemantary Note 2: Information Transfer and Its Relation to Thermodynamics in Complex Dynamical Systems

In this section, we study the relationship between thermodynamics and information transfer by analyzing the effects of information transfer on the thermodynamics of the system. The formulation of information transfer primarily depends on the entropy rates of information-theoretic nature, whereas the study of stochastic thermodynamics relies on entropy production. In [4], the authors study the thermodynamic cost for information flow between two Brownian particles coupled to different heat baths. In their work, the entropy rate is defined as the rate of change of delayed mutual information and is used to formulate the information flow between the two particles. A similar approach that incorporates information theory and the second law of thermodynamics has been proposed recently in [5], where the entropy rate is defined as the time derivative of mutual information and is used to study information flow between two interacting components of a Markovian bipartite system. Below, we show the theoretical relationship between Information transfer and Information flow, thereby providing thermodynamical aspects to the definition of information transfer. We also compare the Information transfer to the widely used Schreiber's transfer entropy. The thermodynamical interpretations of transfer entropy has been studied in [6, 7].

Horowitz Information Flow

The work in [4] provides a thermodynamic formalism that describes the flow of energy and information for a pair of bipartite systems following stochastic thermodynamics. The information flow is defined in terms of the rate of change of mutual information and it modifies the entropy balance of the system. The evolution of the probability distribution is described by a Fokker-Planck equation. We limit our discussion to bipartite systems and assume that the noises in each subsystem X and Y are independent. In [8], the authors extended the thermodynamic formalism information flow to multipartite systems. Specifically, consider a Markovian system (X, Y) with joint probability distribution $p(x, y)$ and evolving according to the equation:

$$\frac{dp(x,y)}{dt} - \sum_{x',y'} \left[W_{x,x}^{y,y'} p(x',y') - W_{x',x}^{y',y} p(x,y) \right] = 0 \tag{25}$$

where $W_{x,x'}^{y,y'}$ defines the transition rate from state (x', y') to (x, y). We assume that the noise in the subsystems X and Y are independent. The current J flowing from (x', y') to (x, y) is defined as

$$J_{x,x'}^{y,y'} = W_{x,x}^{y,y'} p(x',y') - W_{x',x}^{y',y} p(x,y) \tag{26}$$

Combining eq. (25) and eq. (26), we have

$$\frac{dp(x,y)}{dt} - \sum_{x',y'} J_{x,x'}^{y,y'} = 0 \tag{27}$$

Note that eq. (27) is a form of the Fokker-Planck equation in eq. (5), with the noise factor g_{11}, independent of the states. In this approach, the current $J_{x,x'}^{y,y'}$ is split into two separate flows, one flowing from x' to x along y, $J_{x,x'}^{y}$ and the other flowing from y' to y along x, $J_{x}^{y,y'}$. Thus, we have

$$\sum_{x',y'} J_{x,x'}^{y,y'} = \sum_{x'} J_{x,x'}^{y} + \sum_{y'} J_{x}^{y,y'} \tag{28}$$

The joint system (X, Y) satisfies the second law of thermodynamics. Thus the entropy production rate is always positive and satisfies the equation

$$\dot{S}_i = d_t S^{X,Y} + \dot{S}_r \geq 0 \tag{29}$$

where $d_t S^{X,Y}$ is the time derivative of the system's Shannon Entropy, $S^{X,Y}$ and $\dot{S}_r$ is the entropy rate flowing to the environment,

$$\dot{S}_r = \sum_{x \geq x', y \geq y'} J_{x,x'}^{y,y'} \ln \frac{W_{x,x}^{y,y'} p(x',y')}{W_{x',x}^{y',y} p(x,y)} \tag{30}$$

The irreversible entropy production rate is denoted by $\dot{S}_i$. Whereas, eq. (29) describes the flow of entropy between the system and its environment, but it does not explicitly describe the flow of energy and information. The authors pointed out that each term in eq. (29) is a functional of the currents and any current functional, $\mathcal{A}(J) = \sum_{x',y'} J_{x,x'}^{y,y'} A_{x,x'}^{y,y'}$ can be separated into two flows. That is

$$\mathcal{A}(J) = \sum_{x \geq x', y \geq y'} J_{x,x'}^{y} A_{x,x'}^{y,y'} + \sum_{x \geq x', y \geq y'} J_{x}^{y,y'} A_{x,x'}^{y,y'} = \mathcal{A}^X + \mathcal{A}^Y, \tag{31}$$

where $\mathcal{A}^X$ and $\mathcal{A}^Y$ denote the variations in the X and Y directions, respectively. Applying eq. (31) to the second law, we have

$$\dot{S}_i = \dot{S}_i^X + \dot{S}_i^Y, \tag{32}$$

where $\dot{S}_i^X$ and $\dot{S}_i^Y$ are the entropy production rates in each subsystem. The relation between the joint entropy and mutual information, I is given by $S^{XY} = S^X + S^Y - I$, where

$$I = \sum_{x,y} p(x,y) \log \frac{p(x,y)}{p(x),p(y)} \tag{33}$$

The authors define the information flow as the rate of change of mutual information and is given as

$$d_t I = \dot{I}^X + \dot{I}^Y \tag{34}$$

where $\dot{I}^X \geq 0$ signifies that X is learning about Y, thereby increasing the information I and $\dot{I}^X \geq 0$ signifies either consumption of information to extract energy or erasure of information. From eq. (32), eq. (33) and eq. (34), we have

$$\begin{aligned} \dot{S}_i^X &= d_t S^X + \dot{S}_r^{\,X} - \dot{I}^X \geq 0 \\ \dot{S}_i^Y &= d_t S^Y + \dot{S}_r^{\,Y} - \dot{I}^Y \geq 0 \end{aligned} \tag{35}$$

Using eq. (29), eq. (32) and eq. (35), we have

$$d_t S^X + d_t S^Y = d_t S^{XY} + \dot{I}^X + \dot{I}^Y \tag{36}$$

The pair of equations in eq. (35) represents the flow of information derived by the authors. The idea underlying this derivation is the separation of a current functional for a bipartite system into two separate flows.

A Thermodynamic Comparison

We now provide a formal analogy between Horowitz-Esposito's and Liang-Kleeman's definitions of information transfers. By definition, the mutual information, I between two processes, X_1 and X_2 is given by

$$I(t) = \int\int \rho \log\left(\frac{\rho}{\rho_1 \rho_2}\right) dx_1 dx_2 \tag{37}$$

$$I(t) = H_1(t) + H_2(t) - H(t) \tag{38}$$

Differentiating both sides of eq. (39), we have

$$d_t I = d_t H_1 + d_t H_2 - d_t H \tag{39}$$

Using eq. (23) and eq. (39), we have

$$d_t I = T_{1\to 2} + T_{2\to 1} \tag{40}$$

Under the assumption that the noises in each subsystem are independent, eq. (5) and eq. (27) both correspond to the continuity equation. Also, the similarities between the two approaches become self-evident from eq. (23) and eq. (36). Finally, rate of change of mutual information in eq. (34) corresponds to eq. (40). With these analogies, Liang-Kleeman's heuristic approach is supported by the thermodynamics entropic balance approach.

Schreiber's Transfer Entropy

Let P be the probability mass function. The transfer entropy for a Markov chain of order 1, from X_2 to X_1 at time step τ is given by

$$Te_{2\to 1} = \sum P(x_1^{\tau+1}, x_1^\tau, x_2^\tau) \log \frac{P(x_1^{\tau+1} | x_1^\tau, x_2^\tau)}{P(x_1^{\tau+1} | x_1^\tau)} \tag{41}$$

where Te denotes the transfer entropy and it measures the incorrectness when the probability of X_1 at time step τ conditioned on the measurements at previous time steps is taken as the probability of X_1 given the measurements of both X_1 and X_2 at their previous time steps. Notice that eq. (41) can be written as

$$Te_{2\to 1} = \Delta H_1 - \Delta H_{1|2} \tag{42}$$

where

$$\Delta H_1 = -\sum P(x_1^{\tau+1}, x_1^\tau) \log P(x_1^{\tau+1}, x_1^\tau) + \sum P(x_1^{\tau+1}, x_1^\tau) \log P(x_1^\tau) \tag{43}$$

and

$$\Delta H_{1|2} = -\sum P(x_1^{\tau+1}, x_1^\tau, x_2^\tau)\log P(x_1^{\tau+1}, x_1^\tau, x_2^\tau) + \sum P(x_1^{\tau+1}, x_1^\tau, x_2^\tau)\log P(x_1^\tau, x_2^\tau) \tag{44}$$

For ΔH_1, the second term on the right-hand side is

$$-\sum P(x_1^{\tau+1}, x_1^\tau)\log P(x_1^\tau) = -\sum P(x_1^\tau)\log P(x_1^\tau) = H_1(\tau) \tag{45}$$

The first term also has the form of an entropy expression but at a time step between τ and $\tau+1$ and for the time being, we denote it by $H_1(\tau+\frac{1}{2})$. Thus, we can write

$$\Delta H_{1|2} = H_1(\tau+\frac{1}{2}) - H_1(\tau) \tag{46}$$

To see the physical meaning of $\Delta H_{1|2}$, we introduce two quantities

$$A = \sum P(x_1^{\tau+1}, x_1^\tau, x_2^\tau)\log P(x_2^\tau)$$
$$B = \sum P(x_1^\tau, x_2^\tau)\log P(x_2^\tau)$$

It is easy to show that both A and B are equal to $-H_2(\tau)$. Thus, we have

$$\Delta H_{1|2} = -\left[\sum P(x_1^{\tau+1}, x_1^\tau, x_2^\tau)\log P(x_1^{\tau+1}, x_1^\tau, x_2^\tau) + A\right] - \left[-\sum P(x_1^{\tau+1}, x_1^\tau, x_2^\tau)\log P(x_1^\tau, x_2^\tau) + B\right] \tag{47}$$

The last term on the right hand side is the conditional entropy of X_1 on X_2 at time step τ, denoted by $H_{1|2}(\tau)$, while the first term may be interpreted as the conditional entropy of X_1 on X_2 at some time step between τ and $\tau+1$, denoted as $H_{1|2}(\tau+\frac{1}{2})$. So, $\Delta H_{1|2}$ describes the entropy increment of X_1 conditioned on X_2. The two terms correspond to ΔH_1 and $\Delta H_{1\not 2}$ in Liang-Kleeman's formulation. In other words, the freezing of x_2 instantaneously as time goes from τ to $\tau+1$ can be viewed as a kind of conditioning on X_2. The Liang-Kleeman formalism is thus physically consistent with the transfer entropy in the context of a Markov Chain of order 1.

In [9], the authors provide a thermodynamics interpretation of Schreiber's transfer entropy. The authors showed that in a joint system, (X_1, X_2), the entropy variation in each subsystem, ΔS equals the sum of the internal entropy production, σ, and the entropy change caused by the interactions with the surrounding, ΔS_{ext}. That is

$$\Delta S = \sigma + \Delta S_{ext} \tag{48}$$

Further, the authors relate the local transfer entropy to the external entropy production as

$$t_{X_2 \to X_1}(n+1) \propto -\Delta S_{ext}^X \tag{49}$$

where

$$t_{X_2 \to X_1}(n+1) = \log\left[\frac{p(x_1^{n+1}|x_1^n, x_2^n)}{p(x_1^{n+1}|x_1^n)}\right] \tag{50}$$

And the transfer entropy, $Te_{2\to 1}$ is the expected value of $t_{x_2 \to x_1}$ at each time step n. Using eq. (48) for both the subsystems and assuming a proportionality constant equal to 1 in eq. (49), we have

$$\sigma^{X_1} + \sigma^{X_2} = \Delta S^{X_1} + \Delta S^{X_2} + t_{X_2\to X_1}(n+1) + t_{X_1\to X_2}(n+1) \tag{51}$$
$$= \Delta S + t_{X_2\to X_1}(n+1) + t_{X_1\to X_2}(n+1) \tag{52}$$

where $\Delta S = \Delta S^{X_1, X_2}$ in eq. (52). Thus, eq. (52) is analogous to eq. (23) and eq. (36) in Liang-Kleeman's approach and Horowitz-Esposito's approach respectively.

Thus, we provide the thermodynamical and mathematical relationships among the three notions of information-theoretic measures. Moreover, with derivations from the Horowitz-Esposito approach, the heuristic approach in Liang-Kleeman's approach is strengthened. Finally, the thermodynamical relationship between Schreiber's transfer entropy and Liang-Kleeman's information flow is also provided. A rigorous analytical relationship among the three measures is also given in [10].

Supplemantary Note 3: Information Transfer in Linear Stochastic System

Consider a linear time-invariant stochastic network model where the dynamics are given by:

$$dx(t) = Ax(t)dt + B_1 dw(t) \tag{53}$$

where $x(t) \in \mathbb{R}^n$ are the states of the system, $w(t) \in \mathbb{R}^m$ is a white noise with mean zero and unit covariance and B_1 denotes the input noise matrix. We assume that the initial states $x(0)$ denoted as x_0 are drawn from a normal Gaussian distribution, ρ with initial mean, μ_0 and covariance, Σ_0. The non-zero entries of B_1 define how each of the nodes are affected by the white noise.

For the system in eq. (53), the state covariance $\Sigma(t) = \mathbf{E}\{x(t)x(t)'\}$ satisfies the Lyapunov differential equation

$$\dot{\Sigma(t)} = A\Sigma(t) + \Sigma(t)A' + B_1 B_1' \tag{54}$$

Liang-Kleeman's Information Transfer

We use the formulation in eq. (24) to derive the information transfer for the linear time-invariant stochastic system defined in eq. (53). Below, we give an example for a $n = 3$.

Example 1. *For $n = 3$ and initial states drawn from a Gaussian distribution, $\rho_{2|1}$ is a Gaussian with mean $\mu_2 + \sigma_{12}/\sigma_{11}(x_1 - \mu_1)$ and covariance Δ_{12}/σ_{11}. Also,*

$$\rho_{\not{2}} \quad = \quad \rho_{13} \quad = \quad \frac{1}{\sqrt{(2\pi)^2\Delta_{13}}} e^{-\frac{1}{\Delta_{13}}[\sigma_{33}(x_1-\mu_1)^2+\sigma_{11}(x_3-\mu_3)^2-2\sigma_{13}(x_1-\mu_1)(x_3-\mu_3)]} \tag{55}$$

In the above equation, we have used Δ_{ij} to denote $\det \begin{bmatrix} \sigma_{ii} & \sigma_{ij} \\ \sigma_{ij} & \sigma_{jj} \end{bmatrix}$. *Thus, we can write*

$$\int_{\mathbb{R}} \frac{\partial F_1 \rho_{\not{2}}}{\partial x_1} dx_3 = \int_{\mathbb{R}} \rho_{13}\{a_{11} + [\sigma_{13}(x_3 - \mu_3) - \sigma_{33}(x_1 - \mu_1)](a_{11}x_1 + a_{12}x_2 + a_{13}x_3)/\Delta_{13}\}dx_3 \tag{56}$$

$$= a_{11}\rho_1 - \frac{\sigma_{13}\mu_3 + \sigma_{33}(x_1 - \mu_1)(a_{11}x_1 + a_{12}x_2)}{\Delta_{13}}\rho_1 + \frac{1}{\Delta_{13}} \int_{\mathbb{R}} \rho_{13} K dx_3 \tag{57}$$

where $K = a_{13}\sigma_{13}x_3^2 + (a_{11}x_1 + a_{12}x_2)\sigma_{13}x_3 - (\sigma_{13}\mu_3 + \sigma_{33}(x_1 - \mu_1))a_{13}x_3$. Substituting this in eq. (24), we have

$$T^t_{2\to 1} = -E\left[\frac{1}{\rho_1}\frac{\partial F_1 \rho_{13}}{\partial x_1} dx_3\right] = -a_{11} - \frac{1}{\Delta_{13}}[-\sigma_{13}\mu_3 a_{11}\mu_1 - \sigma_{13}\mu_3 a_{12}\mu_2 - \sigma_{33}a_{11}\sigma_{11} - \sigma_{33}a_{12}\sigma_{12}$$
$$+ a_{13}\sigma_{13}\frac{\Delta_{13}}{\sigma_{11}} + a_{13}\sigma_{13}(\mu_3^2 + \sigma_{13}^2/\sigma_{11}^2.\sigma_{11}) + a_{11}\sigma_{13}\mu_3\mu_1 + a_{12}\mu_3\sigma_{13}\mu_2 - a_{13}\sigma_{13}\mu_3^2 + a_{11}\sigma_{13}^2/\sigma_{11}.\sigma_{11} +$$
$$a_{12}\sigma_{13}^2/\sigma_{11}.\sigma_{12} - a_{13}\sigma_{33}\sigma_{13}/\sigma_{11}.\sigma_{11}] = a_{12}\frac{\sigma^t_{12}}{\sigma^t_{11}} \tag{58}$$

Similarly, we can show that for the system in eq. (53) with n random variables,

$$T_{j\to i}(t) = a_{ij}\frac{\sigma_{ij}(t)}{\sigma_{ii}(t)}. \tag{59}$$

Horowitz-Esposito's Information Flow

For the system in eq. (53), the formulation of Horowitz's information flow is given in [10] as

$$\mathcal{T}_{k\to l}(t) = -a_{lk}\frac{C_{lk}(t)}{C_{kk}(t)} - \frac{1}{2}g_{ll}\frac{C_{lk}(t)}{det\Sigma(t)}\frac{C_{lk}(t)}{C_{kk}(t)} \tag{60}$$

where $T_{k\to l}(t)$ is the information flow from x_k to x_l at time t,$a_{ij} = A(i,j)$, $g_{ij} = BB^T(ij)$ and $C_{ij}(t)$ is the (ij) cofactor of the covariance matrix, Σ(t) at time t.

Supplementary Example

For the linear stochastic system in eq. (53), let the constant matrices be

$$A = \begin{bmatrix} -0.2 & 0.5 & 1 \\ -0.2 & 0.4 & -1 \\ -0.5 & 2 & -2 \end{bmatrix}, B1 = \begin{bmatrix} 1 & 0 & 0 \\ 0 & 2 & 0 \\ 0 & 0 & 3 \end{bmatrix}, \Sigma_0 = \begin{bmatrix} 1 & 0 & 0 \\ 0 & 4 & 0 \\ 0 & 0 & 9 \end{bmatrix} \tag{61}$$

where Σ_0 is the initial state covariance matrix. The evolution of the state covariance matrix, $\Sigma(t)$ and the cofactor matrix of $\Sigma(t)$ are shown in Fig.6. The various information transfers among the state variables for the two measures are shown in Fig.7. In the above example, we see that information transfers in both the Liang-Kleeman and Horowitz-Esposito theories approach a constant value after a transition period. The Horowitz information flows in order of magnitude are, $\mathcal{T}_{2\to3}, \mathcal{T}_{1\to3}, \mathcal{T}_{3\to1}, \mathcal{T}_{1\to2}, \mathcal{T}_{2\to1}, \mathcal{T}_{3\to2}$. The Liang-Kleeman information transfers in order of magnitude are, $T_{2\to3}, T_{1\to3}, T_{3\to1}, T_{1\to2}, T_{2\to1}, T_{3\to2}$. Thus, for our example, the two orders are the same. The largest information transfer in both cases is observed in $T_{2\to3}$, as $A(3,2)$ is the largest state matrix element.

Supplementary Note 4: Maximizing Information Theoretic Measures in Linear Stochastic Networks

Below we provide an algorithmic analysis for designing networks that maximize Horowitz's information theoretic measures discussed in the above sections. From eq. (59), $T_{j\to i}$ can be maximized if we can design networks that maximize σ_{ij} and minimize σ_{ii} simultaneously. For Horowitz's information flow, we express eq. (60) in terms of the elements of the state covariance matrix. For example, for networks with 3 nodes, to maximize $\mathcal{T}_{2\to 1}$, we can write

$$\mathcal{T}_{2\to 1}(t) = -a_{12}\frac{C_{12}(t)}{C_{22}(t)} - \frac{1}{2}g_{11}\frac{C_{12}(t)}{det\Sigma(t)}\frac{C_{12}(t)}{C_{22}(t)} \tag{62}$$

For large $\det(\Sigma)$, the second term on the right-hand side of eq. (62) is negligible and thus, we can write

$$\begin{aligned}\mathcal{T}_{2\to 1}(t) &= -a_{12}\frac{C_{12}(t)}{C_{22}(t)}\\ &= a_{12}\frac{(\sigma_{12}\sigma_{33} - \sigma_{23}\sigma_{13})}{\sigma_{11}\sigma_{33} - \sigma_{13}\sigma_{13}}\end{aligned} \tag{63}$$

Also since $\det(\Sigma) \geq 0$, $C_{12}(t) \leq \det(\Sigma)$ and $g_{11} \geq 0$, we get from eq. (62),

$$\mathcal{T}_{2\to 1}(t) \propto a_{12}\frac{(\sigma_{12}\sigma_{33} - \sigma_{23}\sigma_{13})}{\sigma_{11}\sigma_{33} - \sigma_{13}\sigma_{13}} \geq 0 \tag{64}$$

Similarly, to maximize $\mathcal{T}_{3\to 1}$, we have

$$\mathcal{T}_{3\to 1}(t) = -a_{13}\frac{C_{13}(t)}{C_{33}(t)} - \frac{1}{2}g_{11}\frac{C_{13}(t)}{det\Sigma(t)}\frac{C_{13}(t)}{C_{33}(t)} \tag{65}$$

For networks with positive weights,a_{13}, C_{13} and $\det(\Sigma)$ are positive. Therefore, $\mathcal{T}_{3\to 1}$ is always negative. Thus, we maximize the magnitude of $\mathcal{T}_{3\to 1}$. Therefore, similar to eq. (64), we have

$$|\mathcal{T}_{3\to 1}(t)| \propto a_{13}\frac{(\sigma_{13}\sigma_{22} - \sigma_{32}\sigma_{12})}{\sigma_{11}\sigma_{22} - \sigma_{12}\sigma_{12}} \geq 0 \tag{66}$$

From eq. (64) and eq. (66), $|\mathcal{T}_{j\to i}|$ can be maximized by maximizing σ_{ij} and minimizing σ_{ii} simultaneously. The results show that the proposed algorithms in the main manuscript will work for maximizing Horowitz's information flow as well.

Supplementary Note 5: Definitions, Lemmas, Theorems, Propositions, and Proofs

Definition 1. *Walks and paths: In $\mathcal{G}(\mathcal{V},\mathcal{E},\mathcal{W})$, a walk of length k is a sequence of nodes $n_1, n_2, \cdots n_k, n_{k+1}$ such that for all $1 \leq l \leq k$, $(i_l, i_l + 1) \in \mathcal{E}$. A path is a walk with no repeated nodes. The directed graph $\mathcal{G}$ is weakly connected if there is an undirected path between any two nodes.*

Definition 2. *Monotone Increasing: A set function, $f : 2^{\mathcal{E}} \to \mathbb{R}$ is called monotone increasing if for all subsets $P, Q \subseteq \mathcal{E}$, it holds that*

$$P \subseteq Q \implies f(P) \leq f(Q) \tag{67}$$

Definition 3. *Sub-modular, super-modular and modular function: A set function, $f : 2^{\mathcal{E}} \to \mathbb{R}$ is called submodular if for all $P \subseteq Q \subseteq \mathcal{E}$ and $s \in \mathcal{E}\backslash Q$, it holds that*

$$f(P \cup \{s\}) - f(P) \geq f(Q \cup \{s\}) - f(Q) \tag{68}$$

If $-f$ is a sub-modular function, then f is called a super-modular function. If the right and the left hand side of eq. (68) are equal for all P, Q, then the function is said to be modular.

Definition 4. *Communicability : The communicability from node i to node j in $\mathcal{G}(\mathcal{V},\mathcal{E})$, $i, j \in \mathcal{V}$, denoted as $C_c(i,j)$ is defined as the total number of walks of all lengths from node i to j, weighting walks of length k by a factor $\frac{1}{k!}$. It quantifies the ability to exchange messages between two nodes and is given by*

$$\begin{aligned} C_c(i,j) &= [e^{(\mathcal{A}_{0,1})}]_{ij} \\ &= \mathcal{A}_{0,1}(i,j) + \frac{(\mathcal{A}_{0,1})^2}{2!}(i,j) + \cdots \end{aligned} \tag{69}$$

Definition 5. *Submodularity Ratio (γ): For a given non-negative set function f, the submodularity ratio is the largest $\gamma \in \mathbb{R}^+$ such that*

$$\sum_{\omega \in \Omega \backslash S} \Delta_\omega(S) \geq \gamma \Delta_\Omega(S), \quad \forall\ \Omega, S \subseteq \mathcal{E} \tag{70}$$

Definition 6. *Curvature (α): For a given non-negative set function f, the curvature is the smallest $\alpha \in \mathbb{R}^+$ such that*

$$\Delta_j(S\backslash j \cup \Omega) \geq (1-\alpha)\Delta_j(S\backslash j), \quad \forall\ \Omega, S \subseteq \mathcal{E}, \forall j \in S\backslash\Omega \tag{71}$$

Lemma 1. *For the system in eq. (53) with the associated weighted network $\mathcal{G}_{\mathcal{A}}(\mathcal{V},\mathcal{E}_{\mathcal{A}}, w_{\mathcal{A}})$, the elements of Σ in eq. (75) are monotone non-decreasing functions of the edges.*

Proof. We denote Σ as a function of subset A by Σ_A. For all $A \subseteq B \in \mathcal{E}$, $\Sigma(i,j)$ is monotone increasing if $\Sigma_A \preccurlyeq \Sigma_B$ ($\preccurlyeq$ denotes that $[\Sigma(A)](i,j) \leq [\Sigma(B)](i,j)$ for all $(i,j) \in \mathcal{E}$). Since $A \subseteq B$, we take $B = A + \delta A$. Thus, we have

$$\begin{aligned} \Sigma_A(t) = (I + At + \frac{A^2t^2}{2!} + \cdots)\Sigma_0(I + A't + \frac{A'^2t^2}{2!} + \cdots)+ \\ \int_0^t (I + A\tau + \frac{A'^2\tau^2}{2!} + \cdots)B_1B_1'(I + A\tau + \frac{A'^2\tau^2}{2!} + \cdots)d\tau \end{aligned} \tag{72}$$

$$\begin{aligned} \Sigma_{A+\delta A}(t) = (I + (A+\delta A)t + \frac{(A+\delta A)^2t^2}{2!} + \cdots)\Sigma_0 \\ (I + (A+\delta A)'t + \frac{(A+\delta A)'^2t^2}{2!} + \cdots)+ \\ \int_0^t (I + (A+\delta A)\tau + \frac{(A+\delta A)'^2\tau^2}{2!} + \cdots)B_1B_1' \\ (I + (A+\delta A)\tau + \frac{(A+\delta A)'^2\tau^2}{2!} + \cdots)d\tau \end{aligned} \tag{73}$$

Thus,$\Sigma_{A+\delta A}(t) = \Sigma_A(t) + \zeta(A, \delta A, t)$ where $\zeta(A, \delta A, t)$ is an appropriate matrix function of A and δA. Since the entries in δA and A are non-negative, $\zeta(A, \delta A, t) \succcurlyeq 0$. Thus,

$$\Sigma_{A+\delta A}(t) \succcurlyeq \Sigma_A(t) \implies \Sigma_B(t) \succcurlyeq \Sigma_A(t)$$

Theorem 1. *For the system in eq. (53), if we assume that there are no incoming edges to node i except from node j, then $T_{j\to i}$ is a monotone non-decreasing function of edges.*

Proof. For the system in eq. (53), the state covariance $\Sigma(t) = \mathbf{E}\{x(t)x(t)'\}$ satisfies the Lyapunov differential equation

$$\dot{\Sigma}(t) = A\Sigma(t) + \Sigma(t)A' + B_1B_1' \tag{74}$$

The positive definiteness of $\Sigma(t)$ can be seen from the solution of eq. (74) for $\Sigma(0) > 0$, which can be written as

$$\Sigma(t) = e^{At}\Sigma(0)e^{A't} + \int_0^t e^{A\tau}B_1B_1'e^{A'\tau}d\tau \tag{75}$$

The evolution of $\Sigma(t)$ depends on the B_1 matrix which is assumed to be known. If we denote the (i,j) element of $e^{\mathcal{A}_{0,1}}$ by c_{ij}, then we have

$$\left(e^{\mathcal{A}_{0,1}'}\Sigma(0)e^{\mathcal{A}_{0,1}}\right)(i,j) = \begin{bmatrix}c_{1i} & c_{2i} & \cdots c_{ni}\end{bmatrix}\Sigma(0)\begin{bmatrix}c_{1j} & c_{2j} & \cdots c_{nj}\end{bmatrix}' \tag{76}$$

If we take $\Sigma(0) = I_n = B_1B_1'$, from eq. (75), we get

$$\sigma_{ij} = \sum_{k=1}^{n} c_{ki}c_{kj} + \Theta, \quad \sigma_{ii} = \sum_{k=1}^{n} c_{ki}^2 + \Lambda \tag{77}$$

where Θ and Λ are appropriate functions of $c_{ki}c_{kj}$ and c_{ki}^2 respectively. Note that from the definition of communicability (in the main paper), for an incoming edge to node i from p, σ_{pi} increases. Let Δ be the increase in σ_{pi}. Thus, $\Sigma(i,j)$ due to addition of edge (p,i) can be written as

$$\sigma_{ij}' = \sum_{k=1}^{n} c_{ki}c_{kj} + \Theta_1 + \Delta c_{pj} \tag{78}$$

$$\sigma_{ii}' = \sum_{k=1}^{n} c_{ki}^2 + \Delta^2 + 2\Delta c_{pi} + \Lambda_1 \tag{79}$$

where Θ_1 and Λ_1 are appropriate functions of $(c_{ki}+\Delta)c_{kj}$ and $(c_{ki}+\Delta)^2$ respectively. Therefore, the marginal increase in σ_{ii} due to the addition of (p,i) is larger than the increase in σ_{ij} due to the addition of the same edge. Consequently, $T_{j\to i}$ decreases. To avoid this, we fix the in-degree of node i to reduce the number of directed paths to node i. All the remaining paths to node i pass through node j, and by eq. (69), increase in σ_{ij} is greater than increase in σ_{ii}. Hence $T_{j\to i}$ increases.

Corollary 1.1. *Assuming that the elements of $\Sigma(0)$ are positive and $A(i,j) \geq 0$, we can infer from eq. (75) that $\Sigma(t)$ is monotone increasing function of time. Therefore, maximizing the elements of $\Sigma(t)$ at any instant maximizes it for all other instances.*

Theorem 2. *For the linear system in eq. (53), $T_{j\to i}$ is neither a submodular nor supermodular function of the edges.*

Proof. We will prove this with a counter-example. A function is said to be neither a submodular nor a supermodular function if it satisfies the submodularity for some elements and the supermodularity properties for other elements. In this example, we let $B_1 = I_6$, $\mu_0 = 0$, $\Sigma_0 = I_6$. The sets of edges shown by thick lines in Fig. 18a and Fig.18b denote the sets $H = \{G \cup (6,5)\}$ and $I = \{G \cup (4,5)\}$ respectively so that $G \subset H, I$.

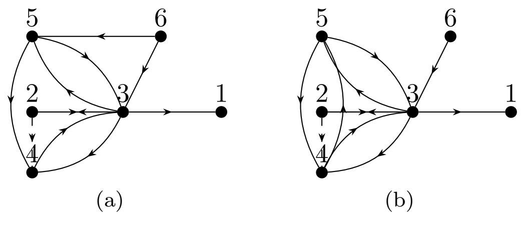

Figure 18: Submodularity and Supermodularity property of $T_{j\to i}$

In Fig. 18a, we compute the following equations

$$T_{3\to1}(G \cup (2,4)) - T_{3\to1}(G) = 0.1156 \tag{80}$$

$$T_{3\to1}(H \cup (2,4)) - T_{3\to1}(H) = 0.1057 \tag{81}$$

Thus, the increase in $T_{3\to1}(G)$ caused by adding edge $(2,4)$ is more than the increase in $T_{3\to1}(H)$ due to the addition of the same edge, which by definition is a submodular function. On the other hand, in Fig. 18b we compute

$$T_{3\to1}(I \cup (2,4)) - T_{3\to1}(I) = 0.1350. \tag{82}$$

Therefore, the increase in $T_{3\to1}(G)$ caused by adding edge $(2,4)$ is lower than the increase in $T_{3\to1}(H)$ due to the addition of the same edge, which by definition is a supermodular function. Thus $T_{3\to1}$ is neither a submodular nor supermodular function of the edges.

Lemma 2. *Maximizing the communicability from all nodes to node j maximizes $T_{j\to i}$.*

Proof. Recall the definition of communicability. The term σ_{ij} is defined in eq. (76) as the communicability from all nodes to node j. Thus, from eq. (59), maximizing σ_{ij} maximizes $T_{j\to i}$.

Theorem 3. *In the ground set $\mathcal{E}_g$, the information transfer function $T_{j\to i}$ is non-negative, non-decreasing and normalised. The bounds on γ and α are given by*

$$\gamma \geq \frac{T_{ji}(\omega_{ij})}{T_{ji}(\mathcal{E}_g) - T_{ji}(\omega_{ij})}, \quad \alpha \leq 1 - \frac{T_{ji}(\omega_{ij})}{T_{ji}(\mathcal{E}_g) - T_{ji}(\omega_{ij})} \tag{83}$$

where $\omega_{ij} = \{(j,i)\}$, $T_{ji} = T_{j\to i}$.

Proof. We first recall the definitions of the submodularity ratio and curvature of a set function. For any $S \subseteq \mathcal{E}_g$, $T_{j\to i}(S)$ is non-negative since the entries of the A matrix are greater than or equal to zero. Also from Theorem 1, $T_{j\to i}$ is a monotone increasing function of the edges. And from eq. (75), $\sigma_{ij} = 0$ when there are no edges in the network. Therefore $T_{j\to i}(\phi) = 0$ and is also normalised. To derive the lower bound of the submodularity ratio, we first derive a lower bound of $\sum_{\omega\in\Omega\backslash S} \rho_\omega(S)$ in eq. (70). Thus, we have

$$\begin{aligned} \sum_{\omega\in\Omega\backslash S} \rho_\omega(S) &= \sum_{\omega\in\Omega\backslash S} f(S\cup\{\omega\}) - f(S); \quad \forall S,\Omega \subseteq \mathcal{E}_g \\ &= \sum_{\omega\in\Omega\backslash S} T_{ji}(S\cup\{\omega\}) - T_{ji}(S) \end{aligned} \tag{84}$$

Note that $T_{j\to i}(S) = 0$ if $(j,i) \notin S$ for any $S \subset \mathcal{E}_g$. Thus for any two disjoint sets $P, Q \subset \mathcal{E}_g$, either $T_{j\to i}(P) = 0$ or $T_{j\to i}(Q) = 0$ or both. Also, since $T_{j\to i}$ is monotone non-decreasing of the edges,

$$T_{j\to i}(P\cup Q) \geq T_{j\to i}(P) + T_{j\to i}(Q)$$

Thus, we can write eq. (84) as

$$\begin{aligned} \sum_{\omega\in\Omega\backslash S} \rho_\omega(S) &\geq \sum_{\omega\in\Omega\backslash S} T_{ji}(S) + T_{ji}(\omega) - T_{ji}(S) \\ &\geq \sum_{\omega\in\Omega\backslash S} T_{ji}(\omega), \end{aligned} \tag{85}$$

where $T_{ji}(\omega) = 0$ if $(j,i) \notin \Omega\backslash S$. Since we are finding the largest γ, we take $(j,i) \in \Omega\backslash S$ and we can write eq. (85) as

$$\sum_{\omega\in\Omega\backslash S} \rho_\omega(S) \geq |\Omega\backslash S| \; T_{ji}(\omega_{ij}) \geq T_{ji}(\omega_{ij}) \tag{86}$$

For the right-hand side in eq.(71), we have $\forall S,\Omega \subset \mathcal{E}_g$,

$$\begin{aligned} \rho_\Omega(S) &= f(S\cup\Omega) - f(S) \\ &= T_{ji}(S\cup\Omega) - T_{ji}(S) \\ &\leq T_{ji}(\mathcal{E}_g) - T_{ji}(\omega_{ij}) \end{aligned} \tag{87}$$

Using the inequalities in eqns. (86) and (87), we can write

$$\gamma \geq \frac{T_{ji}(\omega_{ij})}{T_{ji}(\mathcal{E}_g) - T_{ji}(\omega_{ij})} \tag{88}$$

Similarly, we can bound the curvature. We have $\forall S,\Omega \subset \mathcal{E}_g, k \in S\backslash\Omega$, the left-hand-side of eq. (71) can be written as

$$\begin{aligned} \rho_k(S\backslash j \cup \Omega) &= T_{ji}(S\cup\Omega) - T_{ji}(S\backslash k \cup \Omega) \\ &\geq T_{ji}(S\backslash\omega_{ij} \cup \Omega) + T_{ji}(\omega_{ij}) - T_{ji}(S\backslash\omega_{ij} \cup \Omega) \\ &= T_{ji}(\omega_{ij}) \geq T_{ji}(\omega_{ij}) \end{aligned} \tag{89}$$

For the right-hand side of eq. (71), we have $\forall S, \Omega \subset \mathcal{E}_g, j \in S \backslash \Omega$

$$\begin{aligned} \rho_j(S\backslash j) &= T_{ji}(S) - T_{ji}(S\backslash j) \\ &\leq T_{ji}(\mathcal{E}_g) - T_{ji}(\omega_{ij}) \end{aligned} \tag{90}$$

From eqns. (89) and (90), we have

$$\frac{\rho_j(S\backslash j \cup \Omega)}{\rho_j(S\backslash j)} \geq \frac{T_{ji}(\omega_{ij})}{T_{ji}(\mathcal{E}_g) - T_{ji}(\omega_{ij})} \tag{91}$$

Thus the lower bound for the curvature is

$$\alpha \leq 1 - \frac{T_{ji}(\omega_{ij})}{T_{ji}(\mathcal{E}_g) - T_{ji}(\omega_{ij})} \tag{92}$$

Proposition 1. *Given the optimal edge set S^*,*

i) there always exists a global maximum w^* of Problem 1 and Problem 2 (in the main paper) satisfying

$$\sum_{i=1}^{k} w_i^* = w_{max} \tag{93}$$

ii) if further, $T_{j\to i}$ is not a constant function of w_i for any $i = 1, 2, \cdots k$, then any global maximum satisfies eq. (93). In other words, no global maximum may lie in the interior of the feasibility set $\{0, w_{ub}\}$.

Proof. i) We consider the case when $W_{max} \leq k w_{ub}$ since if $W_{max} > k w_{ub}$, it is trivial to see that $w_i = w_{ub}$. If the global maximum w^* does not satisfy eq. (93), then $\sum_{i=1}^{k} w_i^* < w_{max}$ and we can always find $\hat{w}$ satisfying eq. (93). From Theorem 1 and the fact that w^* is a global maximum, $\hat{w}$ is also a global maximum. Thus $\hat{w} = w^*$.
ii) Let the global maximum be w^*. If $T_{j\to i}$ is not a constant function of w_i and w^* does not satisfy eq. (93), then $\sum_{k=1}^{k} w_k^* < w_{max}$. We define

$$\Gamma_{w^*} = \{w \geq w^* | w \leq w_{ub}, \sum w_i \leq w_{max}\} \tag{94}$$

Let k_{ub} be the number of edges in w that have weights w_{ub}. Since $w \geq w^*$ in Γ_{w^*}, $T_{j\to i}(w) \geq T_{j\to i}(w^*)$. Also, since w^* is the global maximum, $T_{j\to i}(w^*) \geq T_{j\to i}(w)$. Thus $\forall w \in \Gamma_{w^*}$, $T_{j\to i}(w) = T_{j\to i}(w^*) \implies T_{j\to i}$ is a constant function over $\mathbb{R}^{k-k_{ub}}$, which is a contradiction. Hence, the global maximums for both problems satisfy eq. (93).

Algorithms

Greedy Algorithm

Input: $f = T_{j\to i}$, $\mathcal{E}_g$,$\mathcal{E}_b$, k
Output : S^*
Initialization $S \to \phi$, $P \to \phi$
$S \leftarrow S \cup \mathcal{E}_b$, $P \leftarrow P \cup \mathcal{E}_b$
while $|S| < k$ & $P \neq \mathcal{E}_g$ **do**
  $e_i^f = \underset{e \in \{\mathcal{E}\backslash P\}}{\text{argmax}}\, f(S \cup \{e\}) - f(S)$
  $S \leftarrow S \cup e_i^f$,$P \leftarrow P \cup e_i^f$
end while; $S^* \leftarrow S$

Supplementary Note 6: Computational Complexities

We consider the worst-case complexity analysis of the proposed algorithms. We denote β_1 for the cost of computing the set function defined in eq. (59).

The Subgraph Completion Algorithm in Algorithm 1 (of the main paper) runs for n iterations in the worst case. The computational complexity for each iteration is $\mathcal{O}(n^3)$. Hence, the worst-case computational complexity for the Subgraph Completion Algorithm is $\mathcal{O}(n^4)$. The modular addition and complementary modular addition techniques compute $T_{j\to i}$ for each edge and sort them to identify the links with the highest information transfer. The worst-case computational complexity under constant factor approximation for the modular and complementary modular technique is thus $\mathcal{O}(n^2\beta_1+n^4)$. $\mathcal{O}(n^2\beta_1)$ is the cost of computing $T_{j\to i}$ for each possible link, and $\mathcal{O}(n^4)$ is the cost of sorting the links and finding the links with the highest $T_{j\to i}$. For the Greedy Algorithm, the cost of computing $T_{j\to i}$ for each link is $\mathcal{O}(n^2\beta_1)$ and the cost of identifying the link with the highest information transfer is $\mathcal{O}(n^2)$. Thus for each iteration, the total worst-case cost is $\mathcal{O}(n^2\beta_1 + n^2)$. In the worst case, the Greedy Algorithm runs for n^2 iterations. Hence, the worst-case computational complexity is $\mathcal{O}(n^4\beta_1 + n^4)$.

For a network of m nodes, the maximum number of edges is $n = m^2$. The computational cost of $T_{j\to i}$ for $i, j = \{1, 2, \cdots m\}$ involves matrix multiplication and matrix addition of order m. We can approximate the computational complexity of $T_{j\to i}$ as $\beta = \mathcal{O}(m^3)$. For a network of size 10, we plot the computational complexities of our algorithms, which is shown in Figure 8a. The number of edges to be added is usually greater than the network size,i.e., $m < n$. Therefore, further approximations of the computational complexities are given as $\mathcal{O}(n^4)$ for Subgraph Completion Algorithm, $\mathcal{O}(n^5)$ for the modular and the complementary modular technique and $\mathcal{O}(n^7)$ for the Greedy Algorithm. A comparison of complexities as a function of the edges is shown in Figure 8b.

We can see that in both Figures 8a, 8b, the subgraph completion algorithm performs the best. To highlight the differences among these algorithms in terms of network topology, we consider the problem of maximizing $T_{3\to 1}$ in a network of 20 edges by adding 200 edges. We take $B_1 = 0.01I_{20}, \Sigma_0 = I_{20}$, where I_n is the identity matrix of order n. The results are shown in Figures 9, 10.

Figure 9 shows the network topologies generated by Greedy Algorithm, the Subgraph Completion (SC) Algorithm, and the Modular and the Complementary Modular (C-Modular) approaches. To compare the efficiency and the performance of these algorithms, we plot $T_{3\to 1}$ for increasing the number of edges from 0 to 220. As we see from Figure 10, the performances of the Greedy Algorithm and the SC Algorithm are very close, while the Modular and the C-Modular approaches do not perform well. Among all the algorithms, our heuristic SC Algorithm takes has the best computational complexity, and the Greedy Algorithm has the most expensive computational complexity. The simulation justifies the performance of our heuristic SC Algorithm in terms of computational cost and maximizing the objective function. We see similar results in Figure 8 as well.

Supplementary Note 7: Phase Reduction of Coupled Oscillators

To study the oscillatory behaviour of the interacting nodes in complex networks, each nodal dynamic is separated into phases and amplitudes. If the phase-amplitude interactions among the individual nodes are negligible, the phase and amplitude dynamics are decoupled. Thus the network oscillatory dynamics can be expressed in terms of nodal phases only [11]. Throughout this work, we focus on weakly coupled oscillators where the separation between the phase and amplitude dynamics is possible. Consider a dynamical system

$$\dot{x} = f(x) \tag{95}$$

with $x \in \mathbb{R}^n$ and f is a smooth n dimensional vector field. If eq. (95) has a stable limit cycle solution, $x^c(t) = x^c(t+T)$ with time period $T > 0$, then $\dot{x}$ describes oscillatory dynamics. Associating a phase $\phi(t) \in [0, 2\pi)$ described by f on the limit cycle solution, we can write $\dot{\phi} = \omega$, where $\omega = 2\pi/T$. Within the basin of attraction, the phase ϕ can be uniquely defined by assigning to each point x_0 a scalar phase $\phi(x_0)$ such that $\lim_{t\to\infty}\phi[x(t) - (\omega t + \phi(x_0))] = 0$ for the solution $x(t)$ of eq. (95) starting at $x(0) = x_0$. By definition,

$$\frac{d}{dt}\phi(x(t)) = \omega = \nabla_x \phi(x)\frac{dx}{dt} \tag{96}$$

Introducing an additive perturbation, $p(x,t)$ in eq. (95), scaled by a parameter $k \in \mathbb{R}$, eq. (95) can be written as

$$\dot{x} = f(x,p) = f(x) + \partial_p f(x,0)p + \mathcal{O}^2(p) \tag{97}$$

From eq. (96) and (97), for infinitesimal perturbations, $|p| << 1$ and $k = \epsilon << 1$,

$$\frac{d}{dt}\phi = \omega + k\nabla_x \phi(x^c(\phi))p(x^c\phi, t) = \omega + \epsilon z(\phi)p(x^c(\phi), t) \tag{98}$$

In other words, the response in the phase variable ϕ to weak perturbation p can be approximated by $z(\phi)p(x^c(\phi), t)$. Here, $z(\phi)$ is the 'Phase Response Curve (PRC)' and determines the linear response in the phase variable due to small, pulse-like perturbations in the n different coordinates applied at the oscillator's phase ϕ. The oscillatory dynamics are thus reduced to a one-dimensional phase model. Various methods to determine $z(\phi)$ are given in [12]. We employ the adjoint method to determine the PRC.

Phase Reduction for a network of N coupled limit-cycle oscillators under external forcing

Consider a network of $N \in \mathbb{N}$ coupled stable limit cycle oscillators evolving according to

$$\dot{x}_i = f_i(x_i) + \sum_j g_{i,j}(x_i, x_j) + \sum_k h_{i,k}(x_i)\zeta_k \tag{99}$$

with $x_i \in \mathbb{R}^{n_i}, i \in \{1, \cdots N\}$, f_i smooth vector fields, g_{ij} coupling functions, and $h_{i,k}$ denotes the impact of the random processes ζ_k with zero mean. We also assume that the nodal dynamics in the deterministic case $(g_{ij} = 0, h_{i,k} = 0)$ has an attracting limit cycle. Using eq. (98), the reduced phase dynamics of eq. (99) under the assumptions that the couplings and the external forces(noise) are sufficiently weak, can be written as

$$\frac{d}{dt}\phi_i = \omega_i + \sum_j z_i(\phi_i)g_{i,j}(\phi_i, \phi_j) + \sum_k z_i(\phi_i)h_{i,k}(\phi_i)\zeta_k \tag{100}$$

where ω_i is the constant oscillation frequency and $z_i(\phi)$ is the n_i-dimensional PRC. Assuming that the system has a collective deterministic state rotating at an average frequency, $\Omega = \frac{2\pi}{T}$, then the coordinates in eq. (100) may be transformed in terms of φ as $\varphi = \phi_i - \Omega t$. The weak couplings and external forces ensure that the evolution of φ is slow as compared to the collective oscillation. The average change over one period can then be written as

$$\frac{d}{dt}\varphi_i = \omega_i - \Omega + \sum_j \frac{1}{T}\int_0^T z_i(\varphi_i + \Omega t)g_{i,j}(\varphi_i + \Omega t, \varphi_j + \Omega t)dt + \sum_k \frac{1}{T}\int_0^T z_i(\phi_i + \Omega t)h_{i,k}(\varphi_i + \Omega t)\zeta_k dt \tag{101}$$

The first sum term in eq. (101) depends on the phase differences and can be written as $\sum_j \gamma_{ij}(\phi_i - \phi_j)$ where

$$\gamma_{ij}(\varphi_i - \varphi_j) = \gamma_{ij}(\phi_i - \phi_j) = \frac{1}{2\pi}\int_0^T z_i(\phi_i - \phi_j + \psi).g_{i,j}(\phi_i - \phi_j + \psi, \psi)d\psi \tag{102}$$

If we assume that the noises affecting the oscillator dynamics are uncorrelated, that is ${}_{i,k}(\phi) = \delta_{ik} h_i(\phi)$, then the second sum can be written as $\varsigma_i \xi_i$, where

$$\varsigma_i^2 = \frac{1}{2\pi}\int_0^{2\pi} z_i(\psi)h_i(\psi)h_i(\psi)^T z_i(\psi)^T d\psi \tag{103}$$

and $\xi_i = \frac{d\omega_i}{dt}$. Thus the stochastic oscillatory stochastic evolution in eq. (99) can be reduced to the averaged phase stochastic dynamics as

$$\frac{d}{dt}\phi_i = \omega_i + \sum_j \gamma_{ij}(\phi_i - \phi_j) + \varsigma_i \xi_i \tag{104}$$

The study of the effect of network edges on the information flows in complex oscillatory networks is studied using eq. (104) and is provided in the next section.

Computing the Information Transfer in Stochastic Oscillatory Networks

Consider a network of $N \in \mathbb{N}$ coupled stochastic oscillators with phases ϕ_i and intrinsic oscillation frequencies ω_i. Using eq. (104), the stochastic evolution is given by

$$d\phi_i = \left(\omega_i + \sum_j \gamma_{ij}(\phi_i - \phi_j)\right)dt + \sum \varsigma_{ik} dw_k \tag{105}$$

where $\gamma_{ij}(\phi_i - \phi_j)$ are the coupling functions, and external input are modeled as independent Wiener processes, w_k. We assume that in the unperturbed system ($\varsigma_{ik} = 0$), the phase dynamics in eq. (105) has a stable phase-locked state with constant phase difference $\Delta\phi_{ij} = \phi_i^{ref} - \phi_j^{ref}$ and a collective oscillation frequency Ω, that is for all $i \in \{, \cdots N\}$, we have

$$\Omega = \omega_i + \sum_j \gamma_{ij}(\Delta\phi_{i,j}) \tag{106}$$

We decompose the phase dynamics into a deterministic reference part, ϕ_i^{ref} and a fluctuating part, ϕ_i^{fluc}. The solution to the deterministic dynamics is given by

$$\phi_i^{ref}(t) = \Omega t + \Delta\phi_{i,1}^{ref} \tag{107}$$

Introducing new coordinates, $\varphi_i = \phi_i - \phi_i^{ref}$, eq. (105) can be written as

$$d\varphi = f(\varphi)dt + \varsigma dw \tag{108}$$

where $f_i(\varsigma) = \omega_i + \sum_j \gamma_{ij}(\varsigma_i - \varsigma_j + \Delta\phi_{i,j}^{ref}) - \Omega$. We assume that the noise levels, ς_{ik} are small and using the small noise expansion, the first order approximation of eq. (108) is given by a multivariate Ornstein-Uhlenbeck process

$$d\varphi = G\varphi dt + \varsigma dw \tag{109}$$

where $G_{ij} = \gamma_{ij}'(\Delta\phi_{i,j}^{ref})$. Thus, eq. (109) is of the form of eq. (53) and $T_{j\to i}(t)$ has been provided in eq. (59). Thus, the primary goal is to find the coupling matrix, G from the linearized phase response curves.

Supplmentary Note 8: Wilson-Cowan Model for Networks of Neuronal Populations

The Wilson-Cowan model [13] describes the evolution of the excitatory and inhibitory neurons for networks of coupled neuronal populations. The key parameters in this model are the interconnection weights between each sub-populations (excitatory and inhibitory) and the strength of input to each subpopulation. To add the stochastic external noise, we use the model described in

We consider a neuronal network consisting of 8 excitatory and inhibitory sub-populations whose dynamics are described by the average potential variables v_i and u_i, respectively, as

$$\begin{aligned} \tau \frac{d}{dt} v_i &= v_i + g_{ee} g(v_i) - g_{ie} g(u_i) + 0.75 + \sum_j g_{i,j} g(v_j) + \rho_{e,i} \zeta_{e,i} \\ \tau \frac{d}{dt} u_i &= -u_i + g_{ei} g(v_i) - g_{ii} g(u_i) + \rho_{i,i} \zeta_{i,i} \end{aligned} \tag{110}$$

where τ is the membrane time scale, g_{ee} and g_{ei} are the synaptic connections within the excitatory subgroups and from excitatory to inhibitory subgroups respectively. Similarly, g_{ii} and g_{ie} denotes the synaptic connections within the inhibitory and from inhibitory to exhitatory population, $g(v_i)$ denotes the firing rates of v_i and u_i; $\zeta_{i,i}$ and $\zeta_{e,i}$ are the external white noise with weights $\rho_{i,i}$ and $\rho_{e,i}$; $g_{i,j}$ denotes the directed edge weights in the network.

For all our simulations, we use the parameters in [14] given as $\tau = 1$, $g_{ee} = 15$, $g_{ei} = 15$, $g_{ii} = 5$,$g_{ie} = 15$, $\rho_{i,i} = \rho_{e,i} = 0.001$, the edge weights within the cluster $g_{i,j} = 0.1$ and inter-cluster is given by $g_{i,j} = 0.015$, and $g(v_i) = (1 + \exp(-4.4 v_i))^{-1}$. We also use a stochastic integration time step of $\Delta t = 0.01$.

Supplmentary Note 9: Effects of Noise on the Information Transfer

We investigated the influence of noise on the information transfers in weakly coupled oscillators. One of the main effects of noise on neurons is that it allows them to fire even when the voltage levels are below the subthreshold levels. The addition of zero mean noise can induce the neurons to fire. In weakly coupled oscillatory networks, noise can potentially be a factor in the temporal patterns of phase locking or synchronous states or in deciding the level of couplings among the oscillators. The studies in [15, 16] showed that noise is well-known to affect synchronous dynamics in multiple ways, from decreasing the synchronous under the action of noise to increasing synchronous due to correlated noisy input. The coupling strengths among the oscillators depend on the level of synchronization states; thus, noise is a factor in the information transfers among these oscillators. Below, we discuss the effect of channel noise (multiplicative noise) and additive noise on the information transfers in a network of coupled oscillators. We also analyse the effect of local noise acting at a particular instance for a particular duration. We consider a neurological network model consisting of 2 pairs of excitatory and inhibitory neurons.

Effects of Weak Heterogeneity:

In the noiseless case, the averaged phase stochastic dynamics of the two coupled neurons in Figure 11a can be written as

$$d\phi = \Delta\omega + \gamma(-\phi) - \gamma(\phi) = \Delta\omega + \bar{\gamma} \tag{111}$$

where $\phi = \phi_2 - \phi_1$, $\Delta\omega$ denotes the difference in the intrinsic frequencies caused by the presence of weak heterogeneity and $\bar{\gamma} = \gamma(-\phi) - \gamma(\phi)$ is the antisymmetric curve as in Figure 11b. The fixed points are therefore given by $d\phi = 0 \implies \bar{\gamma} = -\Delta\omega$. The phase-locking states of the neurons change due to the presence of weak heterogeneity. The coupling function curve and its antisymmetric part in the absence of heterogeneity are shown in Figure 11b, where the synchronous and anti-phase states are stable. When heterogeneity is added, the neurons move from either the synchronous or the anti-phase state to a nonsynchronous phase-locked state. For example, when $\Delta\omega < 0$ (neuron 1 is faster than neuron 2), the stable steady-state phase-locked values ϕ will be shifted to the upper left of the synchronous and the left of the anti-phase state, and the unstable non-synchronous state shifted to the upper right, as shown in Figure 11c. Furthermore, if $\Delta\omega$ is increased or decreased further, saddle-node bifurcations occur in which a stable and unstable fixed point collide and annihilate each other.

The effect of weak heterogeneity on the information transfer curve for the network in Figure 11a is shown in Figure 12.

Effects of Additive Noise:

Now, to understand the effect of additive noise in the information transfer function, we consider the average phase stochastic dynamics for three oscillators. That is

$$\frac{d}{dt}\phi_i = \omega_i + \sum_j \gamma_{ij}(\phi_i - \phi_j) + D_i\xi_i \tag{112}$$

where $i, j = 1, 2, 3$ and D_i is the noise intensity level in the oscillator i. The variations of the phase difference $\Delta\phi_{1,2}$ for various levels of noise intensities are plotted in Figure 13. Correspondingly, we observe a variation in the coupling function and the phase response curve as shown in Figure 14.

From Figures 13, 14, we observe that the variations in the coupling function increase with the increase in noise intensities. The results are similar to the studies in [17].

Effects of Multiplicative Noise:

In the presence of multiplicative noise, the excitation level dynamics of the oscillators are of the form

$$\frac{d}{dt}v_i = f(v) + g(v)\xi_i(t), \tag{113}$$

where Here ξ_i is white noise with zero mean and intensity, $D = 0.02$. Figure 15 demonstrates the effect of multiplicative noise on the phase differences. A change in the phase difference induces a change in the coupling function and the stable synchronous states. The result is a variation in the information transfer function.

Figure 15a shows that phase synchronization can be significantly enhanced with weak multiplicative noise. As can be seen, for the system with no multiplicative noise, the phase took a long time to settle near the stable phase-locked state. The system with weak multiplicative noise settles near the stable state faster. The result is also demonstrated in [18, 19], where the authors demonstrated that weak multiplication noise

facilitates synchronization in coupled oscillators. Figure 15b is the case when there is no coupling between the oscillators v_2 and v_3. The oscillators are however coupled indirectly through a multiplicative noise in oscillator v_3. The result shows that even in uncoupled oscillators, the stable synchronization state can be achieved faster when the system is affected by weak multiplicative noise. Figure 15c shows the case where multiplicative noise shifts the stable synchronization state. Similar observations are also noticed in Figures 15a and 15b. As the noise intensity increases, we observe an 'oscillation death' phenomenon [20], where the oscillating components approach a stable rest state.

Effect of Local Noise:

As seen from the effects of additive noise, small intensities of additive noise have little effect on the coupling function. We, therefore, analyse the effect of local noise acting at a particular time by inducing multiplicative noise in the excitation level dynamics. The network for 3 nodes is shown in Figure 16a. As before, we follow the Wilson-Cowan dynamics model in Supplementary Note 8 and adapt the parameters given. We first compute the evolution of the excitation levels for $t = 0 - 10$ secs. Next, we induce a small multiplicative noise of intensity 0.02 at node 1 at $t = 5$ secs. Figure 16b shows the coupling function curve for the noiseless case. Due to the multiplicative noise, the stable phase-locked states shift towards the red dots in Figure 16c. The shift in the stable phase-locked state can also be seen in Figure 17a. As a result, $T_{2\to3}(t)$ increases as shown in Figure 17b.

References

[1] X. S. Liang, “The liang-kleeman information flow: Theory and applications,” *Entropy*, vol. 15, no. 1, pp. 327–360, 2013.

[2] X. San Liang and R. Kleeman, “A rigorous formalism of information transfer between dynamical system components. i. discrete mapping,” *Physica D: Nonlinear Phenomena*, vol. 231, no. 1, pp. 1–9, 2007.

[3] ——, “Information transfer between dynamical system components,” *Physical review letters*, vol. 95, no. 24, p. 244101, 2005.

[4] J. M. Horowitz and M. Esposito, “Thermodynamics with continuous information flow,” *Physical Review X*, vol. 4, no. 3, p. 031015, 2014.

[5] J. M. Parrondo, J. M. Horowitz, and T. Sagawa, “Thermodynamics of information,” *Nature physics*, vol. 11, no. 2, pp. 131–139, 2015.

[6] S. Ito and T. Sagawa, “Information thermodynamics on causal networks,” *Physical review letters*, vol. 111, no. 18, p. 180603, 2013.

[7] M. Prokopenko, J. T. Lizier, and D. C. Price, “On thermodynamic interpretation of transfer entropy,” *Entropy*, vol. 15, no. 2, pp. 524–543, 2013.

[8] J. M. Horowitz, “Multipartite information flow for multiple maxwell demons,” *Journal of Statistical Mechanics: Theory and Experiment*, vol. 2015, no. 3, p. P03006, 2015.

[9] D. Hartich, A. C. Barato, and U. Seifert, “Stochastic thermodynamics of bipartite systems: transfer entropy inequalities and a maxwell’s demon interpretation,” *Journal of Statistical Mechanics: Theory and Experiment*, vol. 2014, no. 2, p. P02016, 2014.

[10] H. Kiwata, “Relationship between schreiber’s transfer entropy and liang-kleeman information flow from the perspective of stochastic thermodynamics,” *Physical Review E*, vol. 105, no. 4, p. 044130, 2022.

[11] B. Pietras and A. Daffertshofer, “Network dynamics of coupled oscillators and phase reduction techniques,” *Physics Reports*, vol. 819, pp. 1–105, 2019.

[12] E. M. Izhikevich, *Dynamical systems in neuroscience.* MIT press, 2007.

[13] H. R. Wilson and J. D. Cowan, “Evolution of the wilson–cowan equations,” *Biological cybernetics*, vol. 115, no. 6, pp. 643–653, 2021.

[14] E. Grannan, D. Kleinfeld, and H. Sompolinsky, “Stimulus-dependent synchronization of neuronal assemblies,” *Neural computation*, vol. 5, no. 4, pp. 550–569, 1993.

[15] A. Ghosh, Y. Rho, A. R. McIntosh, R. Kötter, and V. K. Jirsa, “Noise during rest enables the exploration of the brain’s dynamic repertoire,” *PLoS computational biology*, vol. 4, no. 10, p. e1000196, 2008.

[16] J. Zirkle and L. L. Rubchinsky, “Spike-timing dependent plasticity effect on the temporal patterning of neural synchronization,” *Frontiers in Computational Neuroscience*, vol. 14, p. 52, 2020.

[17] Y.-M. Kang, M. Wang, and Y. Xie, “Stochastic resonance in coupled weakly-damped bistable oscillators subjected to additive and multiplicative noises,” *Acta Mechanica Sinica*, vol. 28, no. 2, pp. 505–510, 2012.

[18] Z. Zhu, G. Ren, X. Zhang, and J. Ma, “Effects of multiplicative-noise and coupling on synchronization in thermosensitive neural circuits,” *Chaos, Solitons & Fractals*, vol. 151, p. 111203, 2021.

[19] J. Kurths and C. Zhou, “Noise-enhanced phase synchronization of weakly coupled chaotic oscillators,” in *2003 International Conference Physics and Control. Proceedings*, vol. 2. IEEE, 2003, pp. 353–357.

[20] A. Koseska, E. Volkov, and J. Kurths, “Oscillation quenching mechanisms: Amplitude vs. oscillation death,” *Physics Reports*, vol. 531, no. 4, pp. 173–199, 2013.
